# c-Fos regulated by TMPO/ERK axis promotes 5-FU resistance via inducing NANOG transcription in colon cancer

**DOI:** 10.1038/s41419-024-06451-w

**Published:** 2024-01-17

**Authors:** Yanping Gui, Xiaoping Qian, Youxiang Ding, Qianqian Chen, Fangyu Ye, Yuting Ye, Yingjian Hou, Jun Yu, Li Zhao

**Affiliations:** 1 Public Experimental Platform, China Pharmaceutical University, Nanjing, 211198 China; 2 Suzhou Hospital, Affiliated Hospital of Medical School, Nanjing University, Suzhou, 215153 China; 3 Department of Pathology, Nanjing Drum Tower Hospital, Affiliated to Medical College of Nanjing University, Nanjing, 210008 China; 4 Jiangsu Cancer Hospital, Nanjing, 210009 China

**Keywords:** Colon cancer, Cancer stem cells, Cancer therapeutic resistance

## Abstract

Acquired drug resistance is one of the most common limitations for the clinical response of colon cancer to 5-Fluorouracil (5-FU)-based chemotherapy. The relevant molecular mechanisms might be diversity, but still not be elucidated clearly. In this study, we aimed to investigate the potential mechanisms of c-Fos, a subfamily of activator protein-1, in 5-FU chemoresistance. We determined that phosphorylated c-Fos promoted colon cancer cells resistance to 5-FU by facilitating the cancer stemness. Mechanically, 5-FU treatment induced autolysosome-dependent degradation of TMPO, which subsequently triggered ERK-mediated phosphorylation of c-Fos. Additionally, c-Fos was found to bind to the promoter of NANOG and phosphorylation of c-Fos at Ser 374 was required for its regulation of NANOG expression. NANOG ablation impaired c-Fos/p-c-Fos induced 5-FU resistance and stemness. Taken together, these findings revealed that TMPO-mediated phosphorylation of c-Fos conferred 5-FU resistance by regulating NANOG expression and promoting cell stemness in colon cancer cells. c-Fos could be as a therapeutic target for colon cancer.

## Introduction

Colon cancer has high rates of morbidity and mortality worldwide, posing a major challenge for oncologists [1, 2]. For patients with colon cancer, particularly those with stage III disease, 5-FU is the most commonly used chemotherapeutic agent and fluorouracil-based chemotherapy has been confirmed to be beneficial for overall survival [3]. However, the development of acquired drug resistance occurs frequently and remains one of the most important impediments to the efficacy of clinical response to fluorouracil-based chemotherapy [4, 5]. Therefore, revealing the underlying mechanisms of drug resistance and overcoming acquired resistance has been a long-term focus in colon cancer interventions.

The mechanisms of acquired drug resistance are complex, including hyperactivation of pro-survival signaling axes and loss of suppressive regulations [6]. In addition, highly tumorigenic cancer stem cells (CSCs) have been implicated as a major cause of acquired drug resistance and poor prognosis, as accumulating evidence has indicated that these cells are more resistant to many common chemotherapeutical agents than non-CSCs [7]. In colon cancer, GSCs are characterized by an increased ability of self-renewal, therapeutic resistance, and metastasis, and have been identified through surface markers such as CD44+, CD166+, ALDH+, CD44v6+ [8]. Colon CSCs are also regulated by multiple signaling pathways, including Wnt/β-catenin pathway, BMP pathway, Notch pathway, sonic hedgehog (Shh) signaling, and others [9]. Consequently, targeting CSCs in colon cancer has emerged as a major clinical strategy to improve the treatment of this disease [10].

Thymopoietin (TMPO), also known as Lamina-associated polypeptide 2α (LAP2α), is an important component of nuclear lamina, which lacks a C-terminal transmembrane domain and is therefore localized in the nucleoplasm [11]. Consequently, TMPO is involved in many aspects of cancer cell function. For example, TMPO has been linked to breast cancer metastasis in clinical samples and tissue culture experiments, and its inhibition has been shown to impede the aggressiveness and metastasis in breast cancer by suppressing epithelial-mesenchymal transition (EMT) [12]. Additionally, TMPO is highly expressed in gastric cancer and is associated with a poor prognosis, and its knockdown has been demonstrated to reduce the proliferation and invasion of gastric cancer cells [13].

As a major member of the proto-oncogene activator protein-1 (AP-1) family, c-Fos, encoded by the FOS gene, is known to regulate key cellular processes such as cell differentiation, proliferation, metastasis, and signal transduction in cancer cells [14–16]. It has been demonstrated that post-translational modifications, such as phosphorylation, are essential for c-Fos activity. ERK1/2 is a major kinase that phosphorylates c-Fos at several sites when it is activated and translocated into the nucleus. This allows c-Fos to function as a member of the dimeric AP-1 transcription factor family, thereby mediating target gene transcription and regulating cellular processes [17]. In our previous study, we found that c-Fos promoted the metastasis and invasion of colon cancer, mediated by the activation of p-c-Fos (Ser 374) through cytokine GDF15 [18]. Moreover, recent findings have indicated that c-Fos is associated with drug resistance. Specifically, in MCF7/ADR cells, which are resistant breast cancer cells, c-Fos is a critical regulator of apoptosis and P-glycoprotein (P-gp) expression, and silencing the expression of c-Fos enhanced the sensitivity of MCF7/ADR cells to ADR, 5-FU and CDDP [19]. c-Fos also contributes to the development of multidrug resistance in HEp-2 laryngeal cancer cells via up-regulating P-gp [20]. Meanwhile, recent studies have found that c-Fos also plays a crucial role in the maintenance of cancer stemness [21, 22]. However, the underlying mechanism of c-Fos regulating colon cancer stemness and drug resistance is still poorly understood.

In this study, we investigated the impact of c-Fos on 5-FU resistance in colon cancer. We found that c-Fos promotes the cell stemness by directly binding to the promoter of NANOG, which leads to increased NANOG expression and consequently confers 5-FU resistance in colon cancer. Knocking down NANOG weakened 5-FU resistance and stemness induced by c-Fos/p-c-Fos. Therefore, targeting cancer stemness by inhibiting the activation of c-Fos might be a potential therapy for colon cancer with 5-FU-resistant phenotype.

## Materials and Methods

### Cell culture

HCT116, SW480, A549, HepG2, BGC-823, MCF-7, and HEK-293T cells were purchased from the Shanghai Institute of Cell Biology, Chinese Academy of Sciences (Shanghai, China). HCT116, SW480, HepG2, MCF-7, and HEK-293T cells were cultured in Dulbecco’s modified Eagle’s medium (Gibco, USA) while A549 and BGC-823 cells were cultured in RPMI-1640 medium (Gibco, USA). All mediums were supplemented with 10% fetal bovine serum (FBS; Wisent, China), 100 U/mL benzyl penicillin, and 100 μg/mL streptomycin. All cell lines were cultured at 37 °C in a humidified atmosphere containing 5% CO_2_.

To establish 5-FU-resistant cell lines, human colon cancer cells HCT116 and SW480 were exposed to gradient concentrations of 5-FU (Sigma, Saint Louis, USA, #F6627) ranging from 5 μM to 2 mM. Initially, a low dose of 5-FU was added to the growth medium and the dead cells were discarded. The surviving cells were then cultured in normal medium without 5-FU until they reached a density of 70-80% confluence, a higher dose of 5-FU was added to the medium.

### Plasmids and siRNAs

Plasmids pLX-304-V5 (#25890), pLX304-FOS-V5 (#59140), and GFP-ERK1 (#14747) were bought from Addgene (MA, USA). Flag-TMPO was purchased from Vigenebio (Shandong, China). FOS mutant plasmids were generated using Fast Mutagenesis Kit (Vazyme, Nanjing, China, #C214-01). siRNAs for TMPO were designed and purchased from Genepharma (Shanghai, China). ShRNAs for FOS and NANOG were constructed in our laboratory by binding oligonucleotide of shRNA targeting to the lentiviral vector pLKO.1 puro (Addgene, #8453) at the AgeI and EcoRI enzyme sites. Oligo sequences were all presented in Table S1.

### MTT assay

MTT was the product of BioFroxx (Einhausen, Germany, #1334GR005) and used to determine the viability of cancer cells treated with DMSO or 5-FU for 48 h. The inhibition rate (%) was calculated by [(A_control_- A_treated_)/A_control_] × 100%. A_treated_ and A_control_ were the average absorbances of at least three parallel experiments from treated and control groups, respectively. The resistance index (RI) was computed as the ratio of the IC50 of resistant cells to IC50 of the parental cells, in order to evaluate the resistance of colon cancer cells to 5-FU.

### Cell apoptosis assay

After treatment cells with DMSO or 5-FU for 48 h, cell apoptosis was detected using the Cell Apoptosis Detection Kit (Vazyme, #A211-01) as per the manufacturer’s instructions. Data acquisition and analysis were performed with a flow cytometer (BD Accuri C6, BD Biosciences, New Jersey, USA).

### Colony formation assay

Colon cancer cells were seeded into 12-well plates at an initial density of 1000 cells per well. Following a 10-day treatment with 5-FU, cell colonies were fixed with methanol for 5 mins. Subsequently, 0.1% crystal violet was applied to stain the cells for 15 mins. Finally, the cell colonies were washed with pure water and photographed with a camera.

### CD44 and CD166 detection

Cancer cells were harvested, washed twice with PBS and centrifuged at 2000 rpm for 5 min. 0.5% BSA were used to resuspend cells on ice for 5 min. Then, cell samples were incubated with CD44-FITC (eBioscience, Frankfurt, Germany, #11-0441-81) or CD166-PE (eBioscience, #12-1661-81) antibody at 37 °C for 30 min after centrifugation. Finally, cell samples were detected by flow cytometer (BD Accuri C6, BD Biosciences).

### Sphere formation assay

Colon cancer cells were cultured in the serum-free medium containing 20 ng/mL epidermal growth factor (EGF; Peprotech, Rocky Hill, USA, #100-47) and 20 ng/mL basic fibroblast growth factor (bFGF; Peprotech, #100-18B), 2% B27 supplement (Thermo fisher, Waltham, USA, #17504044). After culturing for 14 days, the number and diameter of spheres were photographed and measured using a Leica DMi8 microscope (Leica, Wetzlar, Germany).

### Western blot

Western blot was performed according to the previous method [18]. Primary antibodies for c-Fos (sc-166940), p-c-Fos (#sc-81485), GFP-tag (sc-9996), V5-tag (sc-81594), and p62 (sc-28359) were purchased from Santa Cruz Biotechnology (Santa Cruz; CA, USA). Primary antibodies for p-ERK1/2 (#4370), LC3 (#4180), CD44 (#37259), K63-linkage ubiquitin (#5621), and β-tubulin (#2148) were obtained from Cell Signaling Technology (CST; Danvers, MA, USA). Primary antibodies for NANOG (A3232), OCT4 (#A7920), SOX2 (#A0561), KLF4 (A13673), Caspase 3 (#A2156), Flag-tag (AE092) were sourced from Abclonal (Wuhan, China). Primary antibodies for TMPO (#14651-1-AP), Lamin A/C (#10298-1-AP), ERK1/2 (#67170-1-Ig), and β-actin (#66009-1-Ig) were acquired from Proteintech (Wuhan, China). Primary antibody for K48-linkage ubiquitin (#ab140601) was purchased from Abcam (Cambridge, UK).

### RT-qPCR assay

TRIzol Reagent (Vazyme, #R401-01) was utilized to extract total RNA according to the manufacturer’s instructions, and concentrations of RNA were determined by NanoDrop 2000 (Thermo Fisher). 1 μg of RNA was reverse-transcribed into cDNA using a reverse transcriptase kit (Vazyme, #R211-01). Quantitative real-time PCR assays were performed using ABI7500 (Applied Biosystems) in 8 strips caps (Axygen, Corning, NY, USA) with SYBR Green PCR Master Mix (Vazyme, #Q331-02). The fold-changes were calculated using the 2^—△△Ct^ method, with glyceraldehyde-3-phosphate dehydrogenase (*GAPDH*) serving as a reference gene. All the primer sequences were presented in Table S2.

### Extraction of cytoplasmic and nuclear fractions

Cytoplasmic and nuclear fractions were extracted according to the previous method [18], and subsequently analyzed by Western blot.

### Electrophoretic mobility shift assay (EMSA)

The chemiluminescent EMSA Kit and biotin-labeled c-Fos probe were purchased from Beyotime Biotechnology (Nanjing, China). Nuclear extracts were prepared following the method described previously [18]. The assay was performed according to the manufacturer’s instruction. The c-Fos probe sequences were detailed in Table S3.

### Immunofluorescence assay

Cells were seeded onto cover glasses in 6-well plate and fixed with 4% paraformaldehyde (PFA) for 15 min, followed by permeabilization with 0.3% Triton-X 100 for 15 min. Then, the cover glasses were blocked by 3% bovine serum albumin (BSA) for 1 h and incubated with primary antibodies at 4 °C overnight. The next day, cells were incubated with Alexa Fluor conjugated secondary antibodies for 1 h and stained with DAPI for 20 min. Confocal microscope (STELLARIS 5, Lecia) was employed to photograph the expressions and location of proteins in cells.

### Co-Immunoprecipitation (Co-IP)

Cell samples were lysed with RIPA buffer (Thermo Fisher, #89901). Subsequently, whole cell lysis was incubated with 1 μg control anti-IgG and 20 μL of Protein A/G PLUS-Agarose (Santa Cruz, #sc2003) at 4 °C for 30 min. After eliminating the beads, 2 μg primary antibody was added to the cell extracts and incubated for 1 h. 20 μL of beads were then added and the mixture was rotated overnight at 4 °C. The following day, the samples were centrifugated at 2500 rpm and the supernatants were carefully discarded. The beads were then washed three times with RIPA buffer, and the samples were mixed with 40 μL of 1×loading buffer and boiled for 10 min. Finally, the samples were analyzed by 10% SDS-PAGE and Western blot as described above.

### Cell transfection and generation of stable cell lines

Plasmid or siRNA was transfected into cells by Lipofectamine 2000 (Invitrogen, Texas, USA, #11668019) for 48 h. HEK-293T cells were transfected with pLX-304-FOS-V5 or shRNAs plasmids, in combination with psPAX2 (Addgene, #12260) and pMD2.G (Addgene, #12259) packing plasmids at a ratio of 4:3:1 to generate lentiviral supernatants. Stable transfected colon cancer cell lines were then obtained according to the method previously described [18].

### Chromatin immunoprecipitation (ChIP) assay

ChIP assay was performed according to the manufacturer’s instruction of ChIP Assay Kit (Beyotime, #P2078). Briefly, cell samples were crosslinked in 4% paraformaldehyde, lysed, and chromatins were sheared into 200–800 bp by ultrasonicator and hybridized with anti-FOS antibody (CST, #2250) or control rabbit IgG for 12 h. Subsequently, the binding complexes were immunoprecipitated with protein A/G agarose for 1 h and then washed with low-salt wash buffer, high-salt wash buffer, TE buffer, and elution buffer. The retrieved DNA samples were reverse cross-linked and purified with a DNA purification kit (Tiangen, Beijing, China, #DP214). For PCR assay, 8 pairs of primers were designed for NANOG promoter region, and the assay was performed using Phanta Max Super-Fidelity DNA Polymerase Kit (Vazyme, #P505-01). All primer sequences are presented in Table S4.

### Luciferase reporter assay

The 2000 bp promoter region of NANOG was cloned into pGL3-Basic Luciferase plasmid (Promega, USA, #E1751) to generate pGL3-Basic-NANOG wild-type plasmid. Subsequently, a pGL3-Basic region-3 deletion of NANOG promoter plasmid was constructed using Fast Mutagenesis Kit (Vazyme, #C214-01). To access the luciferase activity, the Dual Luciferase Reporter Assay Kit (Vazyme, #DL101-01) was employed in HEK-293T cells transfected with the Firefly luciferase and Renilla luciferase plasmids (pRL-TK, Promega, #E2241).

### Immunohistochemistry

Immunohistochemistry assay was performed according to the previous method [18].

### Xenograft tumor model

3 × 10^6^ of HCT116-Vector or HCT116-FOS cells were harvested and injected subcutaneously into the axillae of 5-6 weeks old female BALB/c nude mice, which were purchased from Beijing Vital River Laboratory Animal Technology Co. Ltd. (Beijing, China). The mice were housed under standard specific pathogen-free (SPF) conditions. After 7-10 days, the tumors were measured using a micrometer caliper and the mice were randomly divided into two groups with equal distribution of tumor size around 100 mm^3^ for HCT116-Vector and HCT116-FOS cells, respectively (5 mice/randomized grouping). The mice were then intraperitoneally injected with 0.9% saline or 25 mg/kg 5-FU every 3 days. Tumor volume (TV) was measured every 3 days and calculated using the formula: TV = D/2 × d^2^, where D and d represent the longest and shortest diameters, respectively. After a period of three weeks, the mice were sacrificed and the tumors were extracted, weighed and photographed.

### TUNEL apoptosis assay

TUNEL staining was performed using TUNEL apoptosis detection kit (Vazyme, Cat#A112-01) according to the manufacturer’s instructions. The images were acquired with a fluorescent (DM5200, Leica).

### Patient samples

A total of 22 pairs of paraffin-embedded colon cancer tissues, including initial and recurrent samples, were obtained from patients at Nanjing Drum Tower Hospital (Nanjing, China). The collection of clinical specimens implied with the guidelines in the Declaration of Helsinki and was approved by the Ethics Committee of Nanjing Drum Tower Hospital. Informed consent was obtained from all patients.

### Statistics analysis

Each experiment was repeated at least three times. Data values were analyzed using GraphPad Prism 8 software (GraphPad Software, San Diego, CA). The data were shown as mean ± SD. Comparisons between the groups were estimated by Student’s t-test or Chi-squared test. *p* values < 0.05 were considered statistically significant.

## Results

### TMPO expression was down-regulated and c-Fos was activated in 5-FU-resistant colon cancer cells

**Fig. 1:**
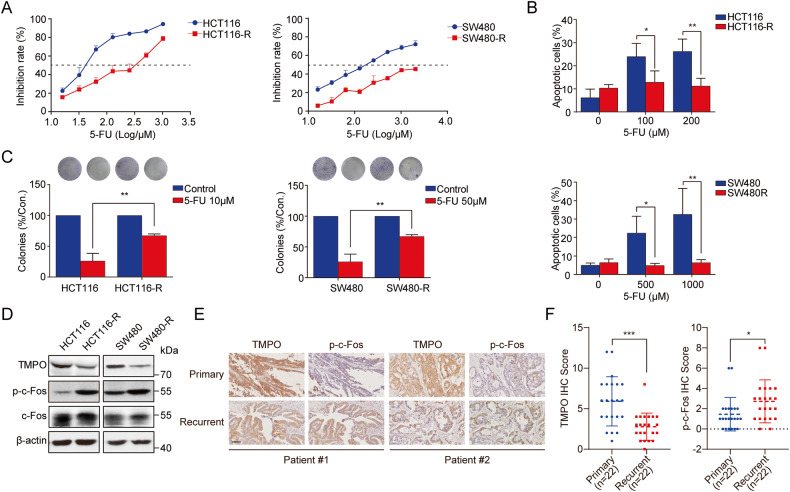
p-c-Fos was elevated in 5-FU-resistance colon cancer cells and negatively correlated with TMPO. **A** Cell viability was detected by MTT assay. **B** Cell apoptosis rates were detected using Annexin-V-FITC/PI apoptosis kit and quantified statistics. **C** Cell proliferation were observed by colony formation assay. **D** TMPO expression and c-Fos phosphorylation were detected via Western blot in both parental and 5-FU-resistant cells. **E** Images of IHC staining of TMPO and p-c-Fos (Ser374) expression in two representative paired primary and recurrent colon cancer tissue. Scale bar = 100 μm. **F** TMPO and p-c-Fos (Ser374) IHC scores of paired primary tumor tissues (*n* = 22) and recurrent tumor tissues (*n* = 22) *P < 0.05, **P < 0.01.

To characterize 5-FU resistance in vitro, we established two 5-FU-resistant colon cancer cell lines (HCT116-R and SW480-R). The resistance index (RI) values were 6.92 and 11.94, respectively, as shown in Fig. 1A. In comparison to parental cells, fewer cell apoptosis was induced by 5-FU in the resistance cells (Fig. 1B). Consistently, the number of colonies of resistant cells was much higher than that of parental cells under the same concentration of 5-FU (Fig. 1C).

Subsequently, we detected expressions of TMPO and c-Fos along with its phosphorylation in 5-FU-resistant cells. Western blot results suggested that compared with parental cells, the expression of TMPO was decreased in 5-FU-resistant cells, while the total expression of c-Fos was not affected, yet its phosphorylation level was significantly increased (Fig. 1D). Moreover, recurrence of tumors is often associated with increased likelihood of developing drug resistance as compared to the initial tumors, so we investigated the expression levels of p-c-Fos and TMPO in 44 colon cancer tissues (22 paired primary and recurrent tissues) and confirmed that recurrent tissues exhibit lower TMPO expression and higher p-c-Fos levels as compared to primary tissues. (Figs. 1E, F). Analysis of correlations between p-c-Fos and clinical parameters revealed that high p-c-Fos was significantly associated with tumor size and low expression of TMPO (Table S5). To better assess the correlation of FOS and TMPO gene expressions with cancer relapse, several GEO datasets were analyzed on the PROGgeneV2 website [23]. Results shown that patients with high expression of FOS had a shorter relapse free survival than those with low expression of FOS (Fig. S1A). Conversely, patients with high expression of TMPO had a longer relapse-free survival than those with low expression of TMPO (Fig. S1B). Meanwhile, those with high expression of FOS/TMPO ratio had a significantly shorter relapse-free survival than those with low expression of FOS/TMPO ratio (Fig. S1C). These results indicated that 5-FU could inhibit TMPO expression and triggered the phosphorylation of c-Fos in colon cancer cells.

### TMPO interacted with ERK1/2 and regulated the phosphorylation of c-Fos

**Fig. 2:**
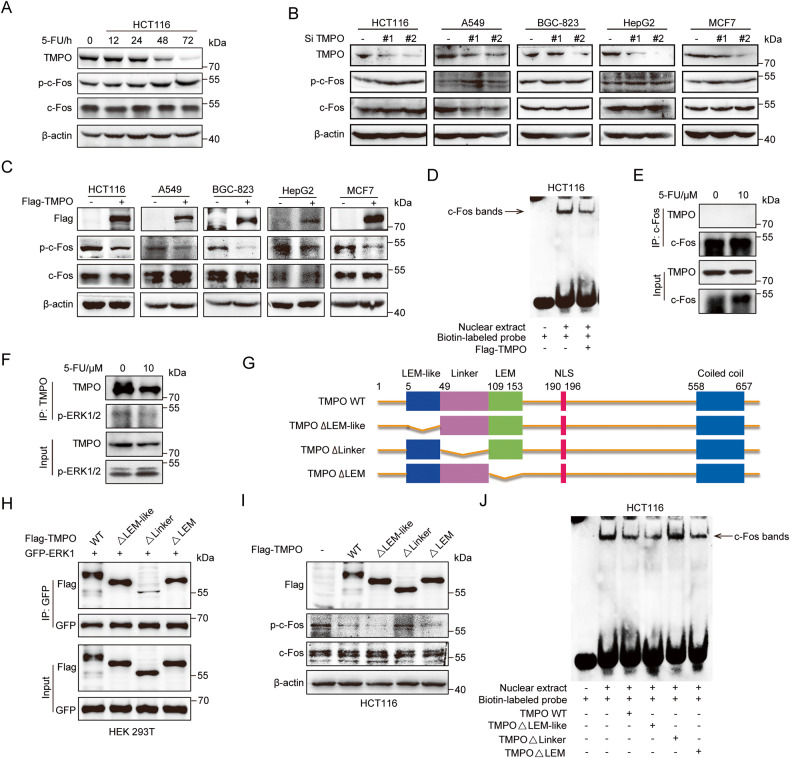
c-Fos phosphorylation was regulated by TMPO via interacting with ERK1/2. **A** After treatment with 5-FU in time-dependent manner, a Western blot assay was performed to analyze TMPO expression and c-Fos phosphorylation in colon cancer cells. **B** After transfection with TMPO siRNAs, several cancer cells were analyzed to investigate the effect of TMPO knockdown on the phosphorylation of c-Fos. **C** In multiple cancer cells, effect of TMPO overexpression on the phosphorylation of c-Fos was confirmed by Western blot. **D** EMSA was conducted to analyze the effect of TMPO overexpression on the transcriptional activity of c-Fos. **E**, **F** Co-IP assay was performed to clarify the interaction of TMPO with c-Fos and p-ERK1/2, respectively. **G** Schematic representation of TMPO protein mutants. **H** Interactions of GFP-ERK1 with TMPO wild-type and mutant plasmids. **I** Determination of p-c-Fos in HCT116 cells transfected with TMPO wild-type or mutant plasmids. **J** Effects of TMPO and its mutants on the transcriptional activity of c-Fos in HCT116 cells.

The administration of 5-FU resulted in a time-dependent decrease in the expression of TMPO and an increase in the phosphorylation of c-Fos in colon cancer cells (Fig. 2A). To further evaluate whether TMPO has an effect on the phosphorylation of c-Fos, we silenced TMPO with siRNAs and exogenously expressed TMPO with plasmid in multiple cancer cells. Western blot analysis indicated that knockdown of TMPO significantly increased the phosphorylation of c-Fos while overexpression of TMPO substantially inhibited the phosphorylation of c-Fos (Fig. 2B, C). Also, EMSA was performed in HCT116 cells transfected with wild-type TMPO plasmids to assess its effect on the transcriptional activity of c-Fos. As shown in Fig. 2D, overexpression of TMPO weakened the DNA binding of c-Fos, which implied that the transcriptional activity of c-Fos was downregulated.

It is reported that TMPO can interact with DNA-binding proteins directly in the nucleus and affect their transcriptional activity [24, 25]. Here, to investigate whether TMPO affects the phosphorylation of c-Fos by protein-protein interaction, Co-IP results were obtained from HCT116 cells. Unexpectedly, the results indicated that TMPO failed to interact with c-Fos (Fig. 2E). Since p-ERK1/2 has been demonstrated to promote the phosphorylation of c-Fos [26–28], we further investigated whether TMPO-regulated c-Fos phosphorylation via interacting with p-ERK1/2. Through Co-IP and immunofluorescence assays, we demonstrated that TMPO could interact with ERK1/2 (Fig. S2A, B). Moreover, the results in Fig. 2F showed that TMPO did interact with p-ERK1/2, while 5-FU inhibited the binding of them, thus might allowing the released p-ERK1/2 to exert its kinase action. To further verify the interaction between TMPO and ERK1/2, a series of Flag-tagged TMPO truncation mutants were constructed (Fig. 2G). As shown in Fig. 2H, deletion of the Linker domain (aa: 49-108) of TMPO almost completely abolished the interaction between ERK1 and TMPO. Additionally, TMPO Linker deletion mutant failed to inhibit c-Fos phosphorylation and transcriptional activity (Fig. 2I, J), indicating that the Linker domain is essential for the function of TMPO. Taken together, these results revealed that the Linker domain of TMPO inhibited the phosphorylation and activation of c-Fos by interacting with ERK1/2.

### Exposure to 5-FU triggered the autolysosome-dependent degradation of TMPO in colon cancer cells

**Fig. 3:**
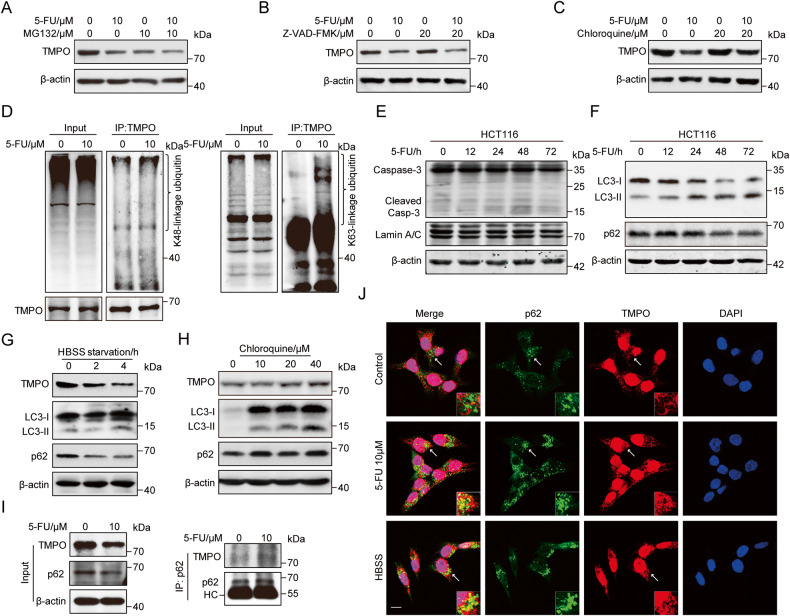
Exposure to 5-FU triggered the autophagy-dependent degradation of TMPO in colon cancer cells. **A**–**C** Inhibitors of three main degradation pathway of eukaryotic proteins, namely MG132, Z-VAD-FMK, and Chloroquine, were used to analyze the effect of 5-FU on the degradation of TMPO in HCT116 cells. **D** K48- and K63-linkage ubiquitin antibodies were used to observe the ubiquitylation of TMPO via Co-IP assay in HCT116 cells. **E** Expressions of cleaved-caspase 3 and its substrate Lamin A/C were detected by Western blot. **F** Expressions of LC3-II/I and p62 were tested via Western blot. **G**–**H** After triggering autophagy with HBSS starvation or inhibiting autophagy with Chloroquine, TMPO expression was detected by Western blot in HCT116 cells. **I** Interaction of TMPO with p62 was determined by Co-IP assay in HCT116 cells. **J** After being treated with 5-FU or HBSS starvation, immunofluorescence assay was employed to detect the co-localization of TMPO and p62 in HCT116 cells. Scar bar= 40 μm.

To investigate the mechanism of 5-FU-induced degradation of TMPO, proteasome inhibitor MG132, lysosome inhibitor chloroquine, and caspase inhibitor Z-VAD-FMK were used. Western blot results revealed that only chloroquine prevented TMPO from 5-FU-induced inhibition (Fig. 3A–C). Since protein ubiquitination is necessary for both proteasome and autolysosome degradation, the ubiquitination type of TMPO triggered by 5-FU in HCT116 cells was examined. Co-IP results indicated that 5-FU treatment augmented the K63-linkage ubiquitination but not K48-linkage ubiquitination of TMPO, which further demonstrated that autolysosome-dependent pathway rather than proteasome pathway was responsible for TMPO degradation (Fig. 3D). In addition, 5-FU treatment did not induce the activation of caspase 3, and the typical substrate Lamin A/C of the caspase degradation pathway did not alter, which further excluded the caspase-dependent degradation pathway (Fig. 3E).

Moreover, it was observed that 5-FU treatment induced the upregulation of LC3-II/I, a well-known marker of autophagosome formation [29], indicating the activation of autophagy. Conversely, the expression of p62, an essential autophagy receptor responsible for delivering cargo to double-membrane vesicles for subsequent autophagic degradation [30], was found be inhibited upon 5-FU exposure (Fig. 3F). Meanwhile, HBSS-induced autophagy strongly lowered the expression of TMPO, while blocking of lysosome by Chloroquine caused a dose-dependent manner upregulation of TMPO expression (Fig. 3G, H). Co-IP experiments further indicated that 5-FU promoted the interaction of TMPO with adaptor protein p62 (Fig. 3I). Immunofluorescence assays also revealed that both 5-FU treatment and HBSS starvation promoted the co-localization of TMPO and p62 in HCT116 cells (Fig. 3J). Collectively, these data illuminated that 5-FU promoted the degradation of TMPO in colon cancer cells via the autophagic lysosomal pathway.

### FOS depletion impairs stemness and 5-FU resistance in colon cancers

**Fig. 4:**
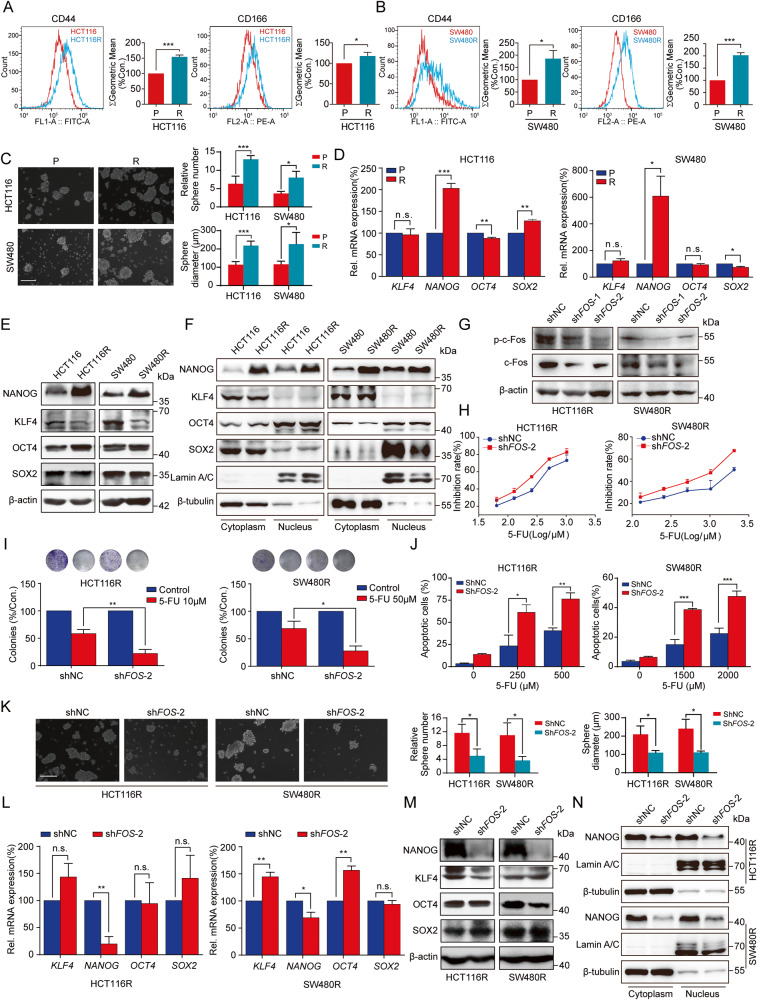
Cell stemness was enhanced in 5-FU-resistant cells and knockdown of FOS sensitized colon cancer cells to 5-FU and down-regulated the expression of NANOG. **A**, **B** Colon CSCs markers including CD44 and CD166 were analyzed by flow cytometer in both parental and resistant cells. **C** Sphere formation of parental and resistant cells. Scale bar = 200 μm. **D** mRNA expressions of stem cell transcription factors including KLF4, NANOG, OCT4, and SOX2 were tested by RT-qPCR. **E** Western blot analyses of protein expressions of the transcription factors. **F** Cytoplasmic and nucleus proteins were analyzed by Western blot. **G** FOS knockdown were detected by Western blot. **H** Cell viability was detected by MTT in resistant cells transfected with negative control or FOS shRNA. **I** Colony formation assay was performed in resistant cells transfected with NC or FOS shRNA. **J** Analysis of the effort of of FOS knockdown on 5-FU resistance by apoptosis assay. Apoptosis rates were calculated respectively. **K** Sphere formation of resistant cells transfected with NC or FOS shRNA. Scale bar = 250 μm. **L** mRNA levels of stem cell transcription factors were detected by RT-qPCR in resistant cells after silencing FOS gene. **M** Western blot analysis of protein expressions of NANOG, KLF4, OCT4, and SOX2. N Effect of FOS knockdown on the entry of NANOG into nucleus was tested by Western blot in resistant cells. n.s.: not significant, *P < 0.05, **P < 0.01, ***P < 0.001.

As cancer stemness has been recognized as a major contributor to chemoresistance in colon cancer, we further detected cancer stemness in both HCT116 and SW480 parental and resistant cells. Flow cytometric analysis showed that expressions of CD44 and CD166, cell surface markers of colon CSCs, were significantly higher in resistant cells than in parental cells (Fig. 4A, B). Consistently, the results in Fig. 4C showed that the sphere formation ability of the resistant cells was much stronger than that of the parental cells, as reflected by the numbers and size of spheres. These data confirmed that 5-FU resistance colon cancer cells possessed a higher cancer stemness than parental cells. The pluripotent transcription factors KLF4, NANOG, OCT4, and SOX2 are essential for maintaining cell stemness in cancer. RT-qPCR and Western blot analysis suggested that compared to parental cells, NANOG expression was remarkably increased in resistant cells, while KLF4, OCT4, and SOX2 expression remained unchanged (Fig. 4D, E). Furthermore, cytoplasmic and nuclear isolation assays reinforced that nuclei translocation of NANOG was significantly higher in resistant cells than in parental cells (Fig. 4F).

To confirm the functional importance of FOS gene in promoting 5-FU resistance, shRNA plasmids were employed (Fig. 4G). As a result, FOS knockdown obviously decreased Fos and p-c-Fos expression and sensitized resistant cells to 5-FU (Fig. 4H–J). Additionally, the sphere formation ability of resistant cells was substantially diminished when FOS was silenced (Fig. 4K). RT-qPCR and Western blot analysis revealed that FOS knockdown inhibited NANOG, but had no effect on KLF4, OCT4, and SOX2 (Fig. 4L, M). Moreover, the translocation of NANOG into nucleus was also largely decreased (Fig. 4N). These results demonstrated that c-Fos could affect colon cancer cell stemness, and the regulation of NANOG might contributed to this process.

### FOS conferred 5-FU resistance via promoting cell stemness in colon cancer cells

**Fig. 5:**
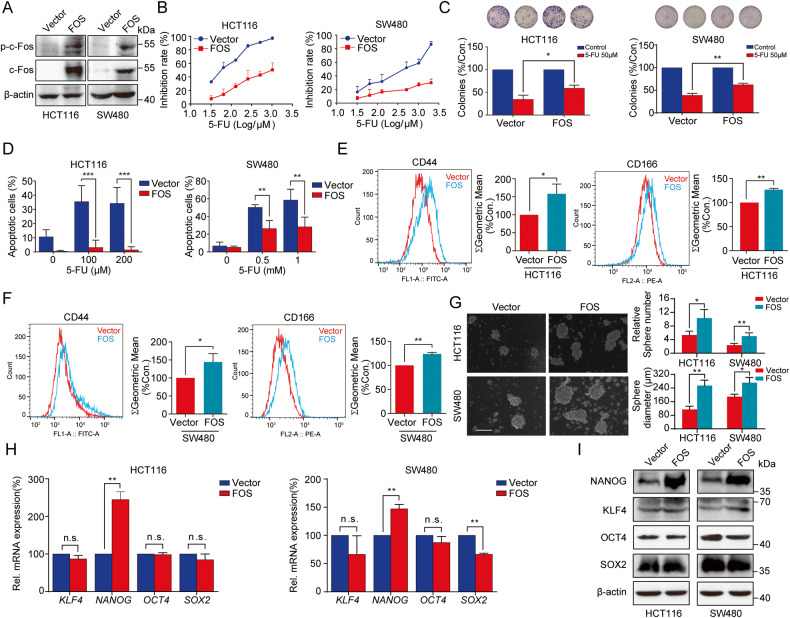
Overexpression of FOS induced 5-FU resistance via promoting the expression of NANOG and increasing stemness in colon cancer cells. **A** Overexpression efficiency of FOS in colon cancer cells was confirmed by Western blot. **B** Cell viability of colon cancer cells with FOS overexpression was detected using MTT assay. **C** Colony formation assay was employed to investigate the effects of FOS overexpression on the proliferation of colon cancer cells after treatment with 5-FU. **D** Effects of FOS overexpression on the 5-FU resistance were analyzed with cell apoptosis assay. Apoptosis rates were counted respectively. **E**, **F** Colon CSCs markers including CD44 and CD166 were detected by flow cytometer in colon cancer cells transfected with FOS or Vector. **G** Sphere formation of colon cancer cells transfected with FOS or Vector. Scale bar= 250 μm. **H**, **I** After transfection with FOS or Vector, colon cancer cells were analyzed by RT-qPCR and Western blot to detect the mRNA and protein levels of KLF4, NANOG, OCT4, and SOX2. n.s., not significant, *P < 0.05, **P < 0.01, ***P < 0.001.

To further interrogate the functional significance of FOS in colon cancer, HCT116 and SW480 cells were stably overexpressed with FOS via lentivirus transduction and the transfection efficiency was verified by Western blot (Fig. 5A). As expected, overexpression of c-Fos/p-c-Fos notably weakened the inhibitory effects of 5-FU on cell viability (Fig. 5B), cell proliferation (Fig. 5C), and cell apoptotic induction (Fig. 5D).

Mechanistically, overexpression of c-Fos was found to promote the expression of colon CSCs markers, including CD44 and CD166, in HCT116 and SW480 cells (Fig. 5E, F). Additionally, sphere formation activity of colon cancer cells was largely strengthened with overexpression of c-Fos (Fig. 5G). RT-qPCR and Western blot analyses further revealed that only the expression of NANOG was impacted by the overexpression of c-Fos (Fig. 5H, I). In sum, these results confirmed that c-Fos was involved in inducing 5-FU resistance by regulating NANOG and augmenting cancer cell stemness in colon cancer cells.

### Phosphorylation of Serine 374 site of c-Fos was indispensable to the regulation of NANOG

**Fig. 6:**
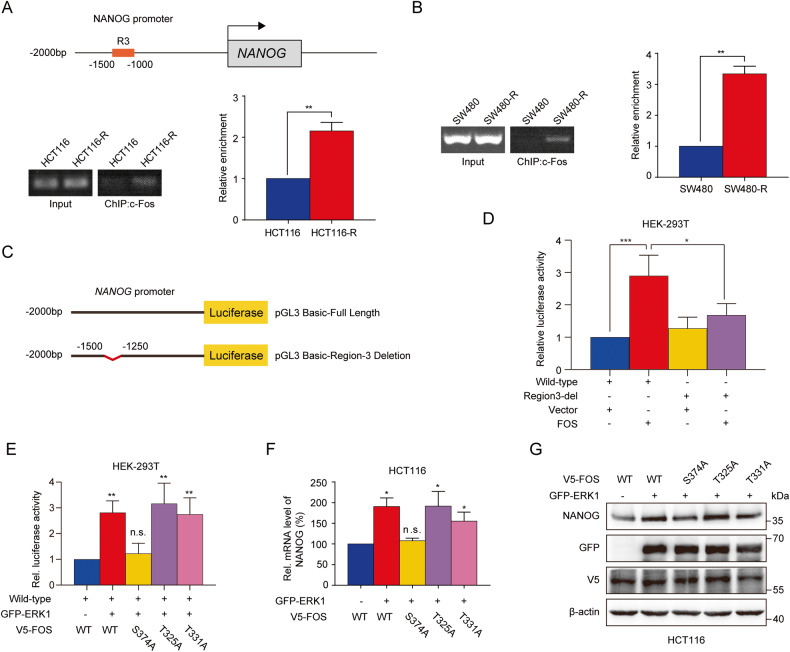
Activation of Serine 374 was indispensable for c-Fos promoting cell stemness of colon cancer through direct regulation of pluripotency regulator NANOG. **A**, **B** ChIP assay was performed to investigate the interaction of c-Fos with NANOG promoter in both parental and resistant cells. The binding fragments of DNA were confirmed by RT-PCR and results were statistically quantified. **C** Schematic representation of luciferase plasmid containing wild-type or mutant promoter sequences of NANOG. **D** Dual luciferase reporter assay was conducted to investigate the binding of c-Fos to wild-type or mutant promoter region of NANOG in HEK-293T cells. **E** Luciferase activity of wild-type FOS and its mutants bounds to the NANOG promoter region in the presence of GFP-ERK1 in HEK-293T cells. **F**, **G** Effect of inactivation of phosphorylation sites of c-Fos on the mRNA and protein levels of NANOG in HCT116 cells co-transfected with GFP-ERK1. n.s., not significant, *P < 0.05, **P < 0.01, ***P < 0.001.

c-Fos was reported to be a transcription factor that associates with c-Jun to form heterodimeric AP-1 complexes that transcriptionally regulate downstream genes [31]. We wondered whether c-Fos drive the expression of NANOG through direct transcriptional regulation. ChIP assay was performed to confirm the interaction of c-Fos with promoter of NANOG in colon cancer cells. 2000 bp promoter region of NANOG (chr12; 7787402 bp to 7789401 bp) were divided into eight fragments, and specific primers were designed, respectively. Figure 6A, B showed that c-Fos specifically bound to region 3 of NANOG promoter, and more c-Fos interacted with region 3 of NANOG promoter in HCT116-R and SW480-R cells compared with parental cells, while no binding was detected in other regions (data not shown). Furthermore, we constructed wild-type and mutant luciferase reporter plasmids containing the NANOG promoter (Fig. 6C). Overexpression of c-Fos remarkably enhanced the luciferase activity of the wild-type plasmid, while only a slight effect on mutant plasmid, which lacks region 3 of the NANOG promoter (Fig. 6D). Therefore, region 3 of NANOG promoter is the specific site that c-Fos bound to.

Studies have reported that the activity of c-Fos is primarily regulated by phosphorylation and that there are three Ser/Thr sites in c-Fos, Ser374, Thr325, and Thr331, which can be phosphorylated by ERK1/2 [26, 32]. To figure out which site is essential for c-Fos to regulate NANOG, we constructed three site-specific mutant plasmids for c-Fos, namely FOS-S374A, T325A, and T331A. Luciferase reporter assay indicated that ERK1 increased the binding of c-Fos to NANOG promoter while no changing in Ser374 mutation group (Fig. 6E). Subsequently, the expression of NANOG mRNA and protein were detected. As shown in Fig. 6F, G, wild-type FOS promoted NANOG expression in the presence of ERK1. For FOS mutants, only FOS-S374A failed to up-regulate NANOG expression. Collectively, these results demonstrated that phosphorylation of Ser374 is essential for c-Fos to regulate the expression of NANOG.

### p-c-Fos/NANOG axis promotes 5-FU resistance and stemness in colon cancers

**Fig. 7:**
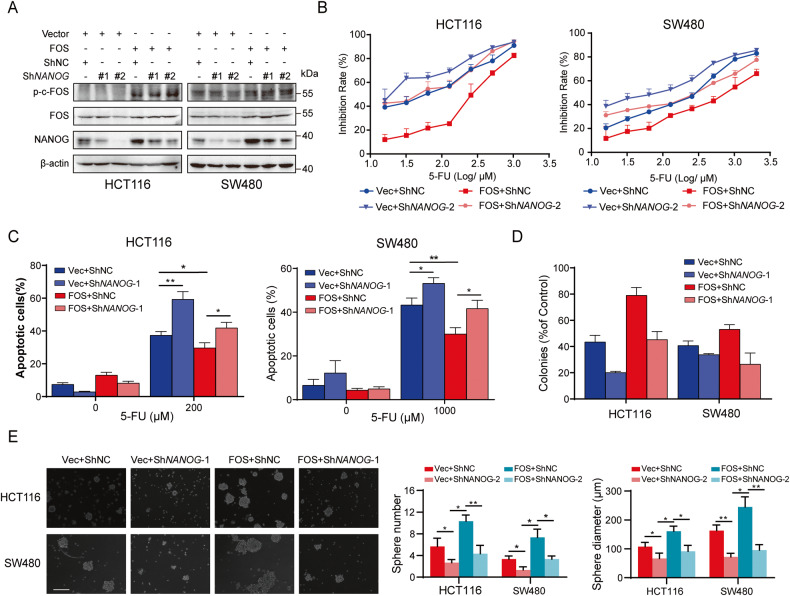
NANOG is required for FOS induced stemness and 5-FU resistance in colon cancer. **A** Vector/FOS-overexpressed HCT116 and SW480 cells were transfected with NC or NANOG ShRNA. p-c-Fos/Fos and NANOG protein expression were detected by Western blot. **B**–**D** Effects of NANOG knockdown on the FOS overexpression induced 5-FU resistance were analyzed with MTT, cell apoptosis and colony formation assays. Apoptosis rates were counted respectively. **E** Sphere formation of Vector/FOS-overexpressed colon cells transfected with NC or NANOG shRNA. Scale bar= 250 μm. n.s., not significant, *P < 0.05, **P < 0.01, ***P < 0.001.

It was then hypothesized that p-c-Fos-mediated transcription and expression of NANOG promote stemness and resistance to 5-FU in colon cancer. We knocked down NANOG expression in HCT116 and SW480 cells overexpressing either a vector or the FOS gene. The results revealed that c-Fos/p-c-Fos functioned as an upstream regulator of NANOG, and knocking down NANOG expression did not significantly affect the expression of c-Fos/p-c-Fos (Fig. 7A). Furthermore, we observed that the increased resistance to 5-FU and stemness induced by FOS overexpression could be reversed by ablating NANOG (Fig. 7B–E). Collectively, these results indicate the essential role of NANOG in mediating the effects of c-Fos/p-c-Fos on promoting stemness and 5-FU resistance in colon cancer.

### Overexpression of FOS conferred resistance of colon cancer to 5-FU in vivo

**Fig. 8:**
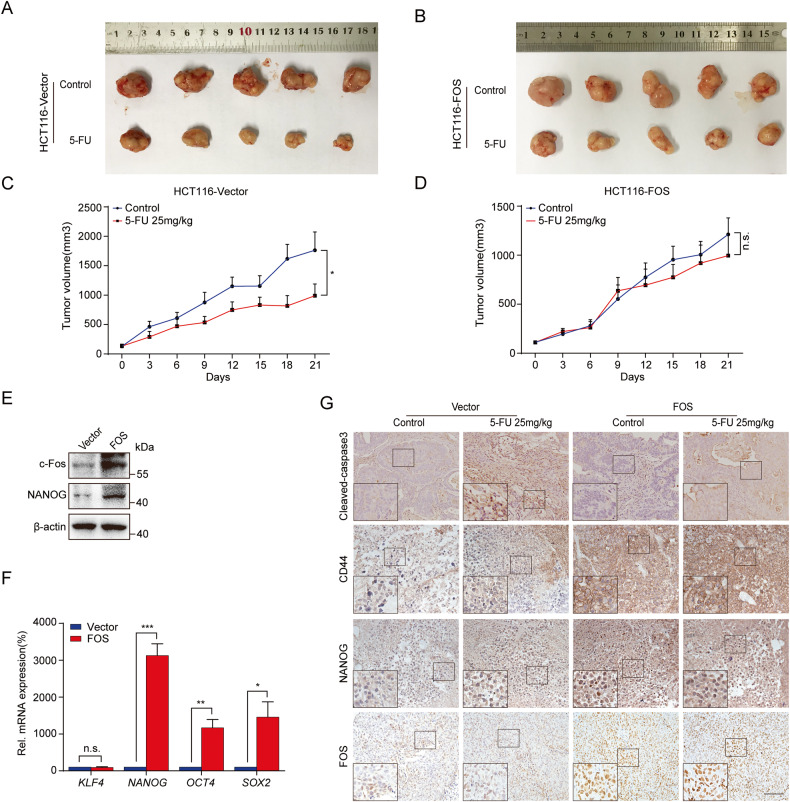
Overexpression of FOS conferred resistance of colon cancer to 5-FU in vivo. **A**, **B** Tumor photos were observed in both HCT116-Vector and HCT116-FOS groups treated with vehicle or 5-FU (*n* = 5 in each group). **C**, **D**) Tumor volumes were quantified statistically (*n* = 5). Tumor volumes were quantified statistically. **E** Western blot analysis of c-Fos and NANOG of the indicated xenografted tumors. **F** Relative mRNA levels of KLF4, NANOG, OCT4, and SOX2 in xenografted tumors of HCT116-Vector and HCT116-FOS. **G** Immunohistochemistry analyses of Cleaved-Caspase 3, CD44, NANOG, and FOS in xenografted tumor tissues of indicated groups. Scale bar = 100 μm. n.s, not significant, *P < 0.05, **P < 0.01, ***P < 0.001.

HCT116-Vector and HCT116-FOS stably transfected cells were injected into the axillae of nude mice in order to establish a xenograft model and to evaluate the effect of c-Fos on the anti-tumor activity of 5-FU in vivo. As shown in Fig. 8A, C, 5-FU significantly inhibited the growth of tumor, with an inhibitory rate of 60%. However, in HCT116-FOS group, 5-FU showed only a 20% suppression (Fig. 8B, D). These results exhibited that overexpression of c-Fos conferred 5-FU resistance to colon cancer in vivo.

Subsequently, tumor homogenates were examined by Western blot and RT-qPCR assays. The exogenous expression of c-Fos triggered an increase in the protein level of NANOG (Fig. 8E). As shown in Fig. 8F, the mRNA expression of NANOG, OCT4, and SOX2, was remarkably up-regulated in the HCT116-FOS group, in which, NANOG was up-regulated most significantly. Furthermore, immunohistochemistry analysis clarified that overexpression of c-Fos increased the CD44 and NANOG expression in HCT116-FOS tissues compared to HCT116-Vector groups, and when 5-FU was administered, Cleaved-Caspase3 expression and the proportion of TUNEL positive cells decreased (Fig. 8G, Fig. S3A). These results demonstrated that overexpression of c-Fos enhanced cancer stemness and 5-FU resistance in vivo.

## Discussion

**Fig. 9:**
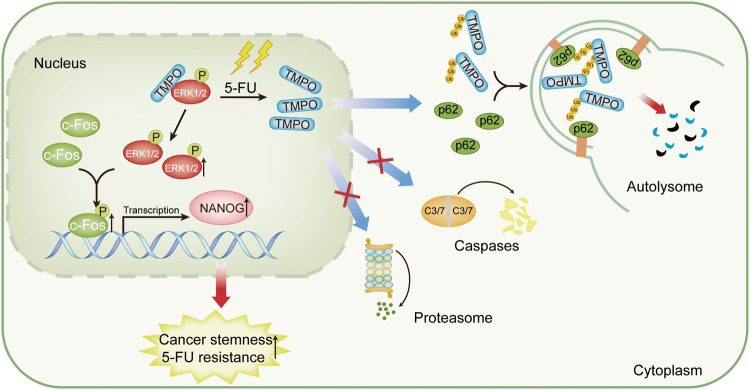
Schematic diagram of the TMPO/c-Fos/NANOG axis promoting stemness and 5-FU resistance in colon cancer. 5-FU treatment promotes the degradation of TMPO through autophagy-related pathway and subsequent activation of c-Fos by ERK1/2, which enhances enhancing cancer stemness and induces resistance to 5-FU in colon cancer by directly regulating NANOG transcriptional expression.

In this study, we found that 5-FU treatment triggers the autophagy-dependent degradation of TMPO and reduces its interaction with ERK1/2 in colon cancer cells. Subsequently, ERK1/2 phosphorylates and activates c-Fos, which increases cell stemness and confers 5-FU resistance through the upregulation of NANOG in colon cancer (Fig. 9). Our work uncovers a novel mechanism and an essential role for c-Fos in the chemoresistance and cancer stemness of colon cancer, suggesting that c-Fos may be a potential target for therapeutic intervention to overcome chemoresistance in colon cancer.

Although colon CSCs comprise only a small proportion of the tumor, they possess the capability to drive tumor progression and chemotherapeutic relapse [8]. Nowadays, various cell surface markers, including CD44, CD166, CD133, ALDH, EphB2, and LGR5, have been used in the identification of colon CSCs [33, 34]. Our results confirmed that expressions of CD44 and CD166 were notably higher in resistant colon cancer cells. Despite the widespread use of these markers to isolate CSCs, conventional chemotherapy generally fails to eliminate the small population of CSCs. Besides, colon CSCs inhabit a functional niche at the base of crypt, which helps to sustain stem cells homeostasis through various signaling pathways [35]. Thus, targeting cancer cell stemness has been an effective treatment strategy for advanced colon cancer [36].

Proto-oncogene FOS has been shown to cause resistance to chemotherapeutic treatment of cancer other. Targeting c-Fos has been demonstrated to abrogate intrinsic resistance to tyrosine-kinase inhibitor therapy in BCR-ABL-induced leukemia [37]. Furthermore, c-Fos has been reported to induce multidrug resistance through up-regulation of p-glycoprotein in many types of cancers [19, 20]. In the present study, we found that overexpression of c-Fos conferred 5-FU resistance in colon cancer cells. Mechanistically, our results unraveled that c-Fos enhanced cell stemness in colon cancer via direct up-regulation of the transcription factor NANOG. Interestingly, other transcription factors including KLF4, OCT4, and SOX2 were unaffected by c-Fos in colon cancer cells. However, overexpression of c-Fos could up-regulate OCT4 and SOX2 expressions in addition to NANOG in xenograft tumors. This may be due to the heterogeneous composition of the tumor microenvironment in xenograft tumors, such as tumor cells, stromal cells, macrophages, cytokines, which may collectively regulate the transcription factors. For example, Yang et al. reports that tumor-associated macrophages increase the expressions of NANOG, OCT4 and SOX2 via EGFR/Stat3 signaling in murine breast cancer cells [38].

The pluripotent transcription factor NANOG is a stem cell marker that has been reported to regulate cell stemness in various cancers [39]. Moreover, studies suggest that NANOG possesses pro-tumorigenic attributes, which promotes tumor development and progression [39–41]. Especially, overexpression of NANOG has been found to predict tumor progression and poor prognosis in colorectal cancer [42, 43]. In combination with KLF4, OCT4, and SOX2, NANOG composed a core set of pluripotent transcription factors that regulate CSCs [44]. Our results uncovered that NANOG, rather than KLF4, OCT4, and SOX2, was significantly up-regulated in FOS-overexpressed colon cancer cells. Mechanistically, c-Fos was found to bind to the promoter region of NANOG and phosphorylation of Ser 374 was determined to be vital for c-Fos to regulate NANOG expression. Our findings clarified a new molecular mechanism of NANOG regulation in colon cancer.

In summary, our results demonstrated that 5-FU treatment promotes the degradation of TMPO through autolysosome-related pathway and subsequent activation of c-Fos through ERK1/2 in colon cancer cells, which disclosed a tight negative correlation between TMPO expression and c-Fos activation. Mechanistically, overexpression of c-Fos was found to induce the resistance to 5-FU in colon cancer cells by augmenting cancer stemness through the direct regulation of the pluripotent transcription factor NANOG. Conversely, knockdown of c-Fos sensitized the resistant cells to 5-FU. Collectively, these results suggest that inhibition of c-Fos activation may be a promising strategy for the treatment of resistant colon cancer.

### Supplementary information

Supplementary Figures and legends

Supplemtentary Tables

Western blot original gels

Reproducibility checklist

## Data Availability

The data presented in this study are all available from the corresponding author upon reasonable request. Publicly available GEO datasets were analyzed in PROGgeneV2 database (http://www.progtools.net/gene/).
