# Supplementary material for: c-Fos regulated by TMPO/ERK axis promotes 5-FU resistance via inducing NANOG transcription in colon cancer
Source: Cell Death Dis. 2024 Jan 17;15(1):61. doi: 10.1038/s41419-024-06451-w (PMC10794174; doi:10.1038/s41419-024-06451-w)
Supplement: Supplementary file 1 — Supplementary Figures and legends [file 41419_2024_6451_MOESM1_ESM.docx]

**Supplementary Figures**


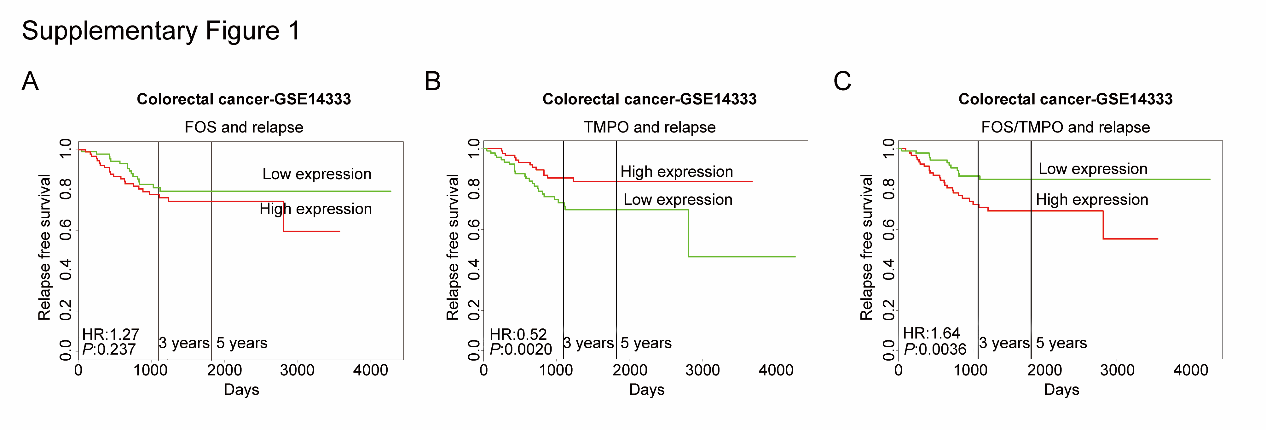


**Figure S1. (A-C)** Relapse free survival curves of colorectal cancer patients with high expression of FOS and low TMPO expression or low expression of FOS and high TMPO expression based on GSE14333 database via PROGgeneV2.


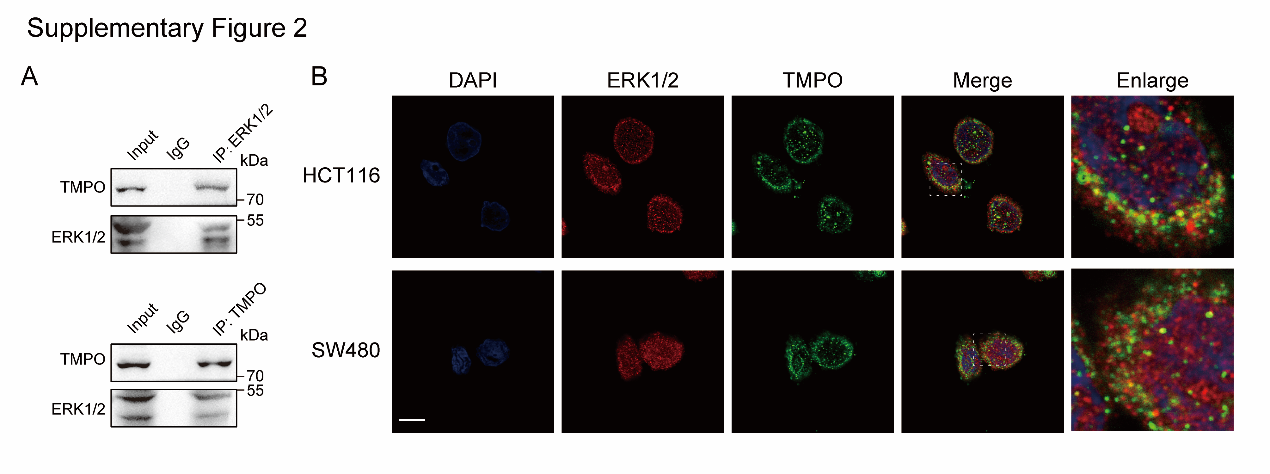


**Figure S2. (A)** Co-IP assays of ERK1/2 and TMPO in HCT116 cells**. (B)** IF assays of ERK1/2 and TMPO in HCT116 and SW480 cells. Scale bar, 10 μm.


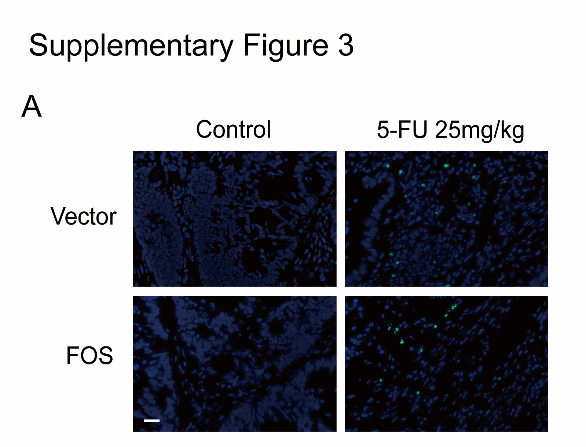


**Figure S3. (A)**. TUNEL assays in xenografted tumors of HCT116-Vector and HCT116-FOS with 5-FU or saline. Scale bar, 25 μm.
