# Supplementary material for: c-Fos regulated by TMPO/ERK axis promotes 5-FU resistance via inducing NANOG transcription in colon cancer
Source: Cell Death Dis. 2024 Jan 17;15(1):61. doi: 10.1038/s41419-024-06451-w (PMC10794174; doi:10.1038/s41419-024-06451-w)
Supplement: Supplementary file 2 — Supplemtentary Tables [file 41419_2024_6451_MOESM2_ESM.docx]

**Table S1. Sequences list for siRNA and shRNA**

| **siRNA** | **Sense (5’ to 3’)** | **Antisense (5’ to 3’)** |
| --- | --- | --- |
| siControl | UUCUCCGAACGUGUCACGUTT | ACGUGACACGUUCGGAGAATT |
| siTMPO-1 | GGUCCUAUUGUGGGAACAATT | UUGUUCCCACAAUAGGACCTT |
| siTMPO-2 | GCUAAGAAAGUACAUACUUTT | AAGUAUGUACUUUCUUAGCTT |
| **shRNA** | **Sense (5’ to 3’)** | **Antisense (5’ to 3’)** |
| Sh-*FOS*-1 | GATCCGGAACAGTTATCTCCAGAAGATTCAAGAGATCTTCTGGAGATAACTGTTCCTTTTTTG | AATTCAAAAAAGGAACAGTTATCTCCAGAAGATCTCTTGAATCTTCTGGAGATAACTGTTCCG |
| Sh-*FOS*-2 | GATCCCTACCTATACGTCTTCCTTTTCAAGAGAAAGGAAGACGTATAGGTAGTTTTTTG | AATTCAAAAAACTACCTATACGTCTTCCTTTCTCTTGAAAAGGAAGACGTATAGGTAGG |
| Sh-NC | GATCCGTTCTCCGAACGTGTCACGTTTCAAGAGAACGTGACACGTTCGGAGAACTTTTTTG | AATTCAAAAAAGTTCTCCGAACGTGTCACGTTCTCTTGAAACGTGACACGTTCGGAGAACG |
| Sh-*NANOG*-1 | CCGGGCTTTGAAGCATCCGACTGTACTCGAGTACAGTCGGATGCTTCAAAGCTTTTTG | AATTCAAAAAGCTTTGAAGCATCCGACTGTACTCGAGTACAGTCGGATGCTTCAAAGC |
| Sh-*NANOG*-2 | CCGGCCTGGAACAGTCCCTTCTATACTCGAGTATAGAAGGGACTGTTCCAGGTTTTTG | AATTCAAAAACCTGGAACAGTCCCTTCTATACTCGAGTATAGAAGGGACTGTTCCAGG |

**Table S2. Primers list for qRT-PCR**

| **Gene** | **Forward (5’ to 3’)** | **Reverse (5’ to 3’)** |
| --- | --- | --- |
| *GAPDH* | AACAGCGACACCCACTCCTC | GGAGGGGAGATTCAGTGTGGT |
| *KLF4* | GCCCCTCGGGCGGCTTCGTGGCCGAGCTC | CGTACTCGCTGCCAGGGGCG |
| *NANOG* | AATACCTCAGCCTCCAGCAGATG | TGCGTCACACCATTGCTATTCTTC |
| *OCT4* | CTTGCTGCAGAAGTGGGTGGAGGAA | CTGCAGTGTGGGTTTCGGGCA |
| *SOX2* | AAATGGGAGGGGTGCAAAAGAGGAG | CAGCTGTCATTTGCTGTGGGTGATG |
| *FOS* | CAGACTACGAGGCGTCATCC | AGTTGGTCTGTCTCCGCTTG |

**Table S3. Sequences list for EMSA**

|  | **Sense (5’ to 3’)** | **Antisense (5’ to 3’)** |
| --- | --- | --- |
| c-Fos probe | CGCTTGATGACTCAGCCGGAA | GCGAACTACTGAGTCGGCCTT |

**Table S4. Primers list for ChIP-PCR**

| **Fragments** | **Forward (5’ to 3’)** | **Reverse (5’ to 3’)** |
| --- | --- | --- |
| *Region 1* | TCACTCCGGAGGCTCTTACC | TTGCAGAAGGGGGTCTTTGC |
| *Region 2* | GACAGGAGGGCAAGTTTTTCC | AGTGGGGCTAGTACATCATGC |
| *Region 3* | TCAGCTTGTGTGGGAGCAAAG | TGACTCATTCTCCTCTGCACTCT |
| *Region 4* | GATGGGCACGGAGTAGTCTT | CTATCCCTCCTCCCAGGTAGT |
| *Region 5* | TGCCTTGGCTTCATGCTAT | GGCAGAAATTTCAACTAGCTCCA |
| *Region 6* | TGGCGCGATATCGGCTCACC | CGGATCACCTGAAGTCAGGA |
| *Region 7* | TCTTCAGGTTCTGTTGCTCGG | TCTACCAGTCTCACCAAGGC |
| *Region 8* | AGGGGTGGGTCTAAGGTGAT | AACATGAGGCAACCAGCTCA |

**Table S5. The correlation between clinicopathological parameters and p-c-Fos protein expression in 44 colon cancers**

| **Parameters** | **Case** | **p-c-Fos expression** | | ***p* value** |
| --- | --- | --- | --- | --- |
|  |  | **Low** | **High** |  |
| **Age (years)** | |  |  |  |
| <60 | 17 | 13 | 4 | 0.4877 |
| ≥60 | 27 | 18 | 9 |  |
| **Gender** |  |  |  |  |
| Male | 22 | 14 | 8 | 0.3216 |
| Female | 22 | 17 | 5 |  |
| **Tumor size (cm)** | |  |  |  |
| <5 | 21 | 20 | 1 | **0.01367** |
| ≥5 | 23 | 15 | 8 |  |
| **Depth of invasion** | |  |  |  |
| T1/T2 | 8 | 6 | 2 | 0.7554 |
| T3/T4 | 36 | 25 | 11 |  |
| **Lymph node metastasis** | | |  |  |
| N0 | 17 | 12 | 5 | 0.9877 |
| N1/N2/N3 | 27 | 19 | 8 |  |
| **TMPO expression** | |  |  |  |
| Low | 19 | 10 | 9 | **0.02388** |
| High | 25 | 21 | 4 |  |
