# Supplementary material for: c-Fos regulated by TMPO/ERK axis promotes 5-FU resistance via inducing NANOG transcription in colon cancer
Source: Cell Death Dis. 2024 Jan 17;15(1):61. doi: 10.1038/s41419-024-06451-w (PMC10794174; doi:10.1038/s41419-024-06451-w)

Full unedited gels for Figure 1D, 2A and 2B

Figure 1D

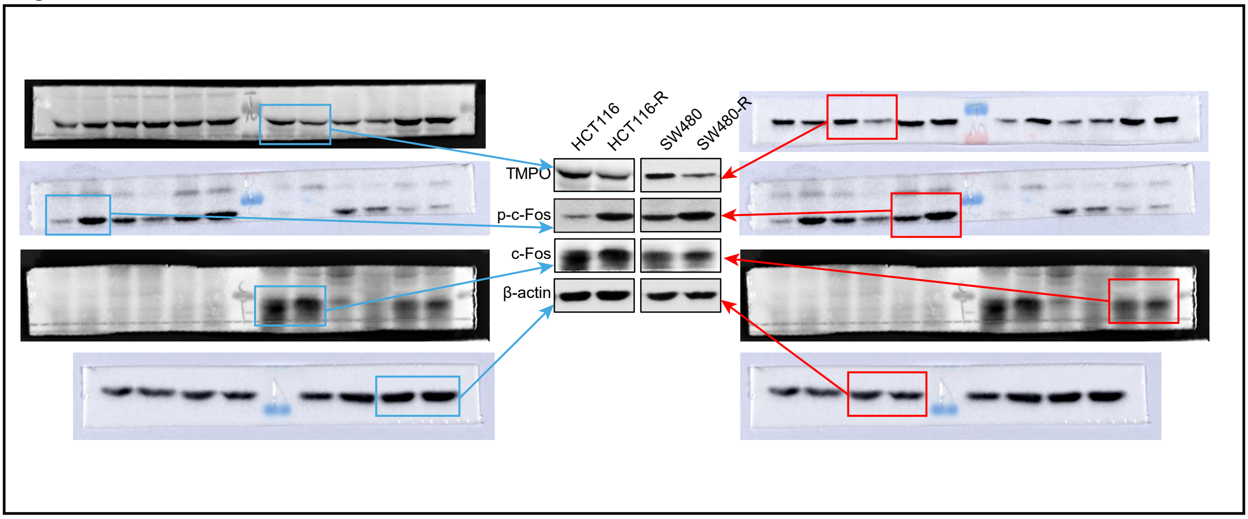

Figure 2A

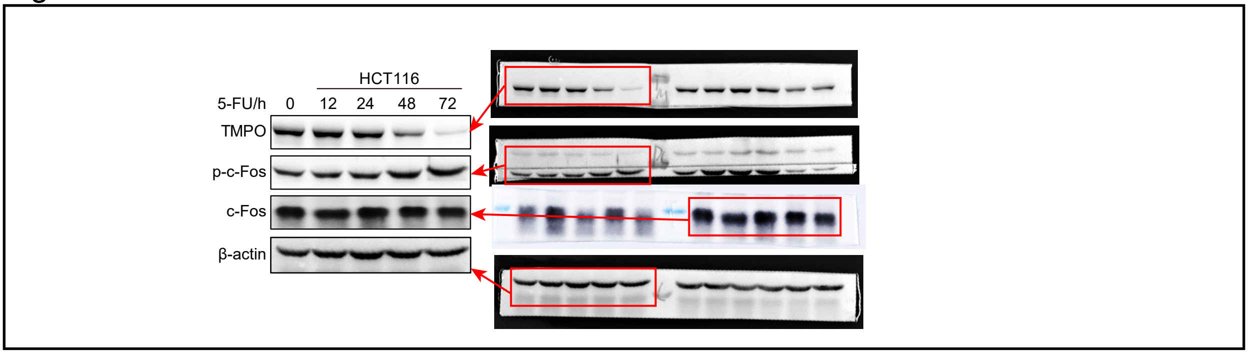

Figure 2B

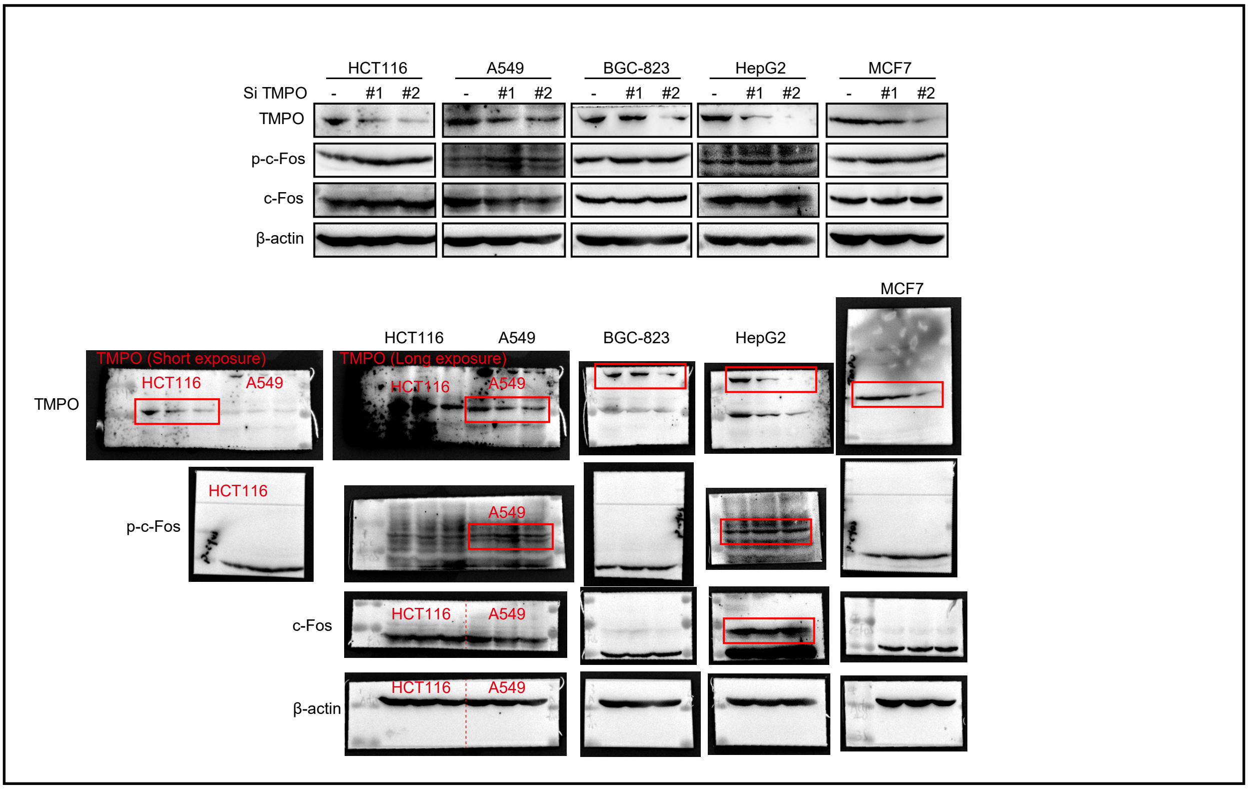

Full unedited gels for Figure 2C, 2D, 2E and 2F

Figure 2C

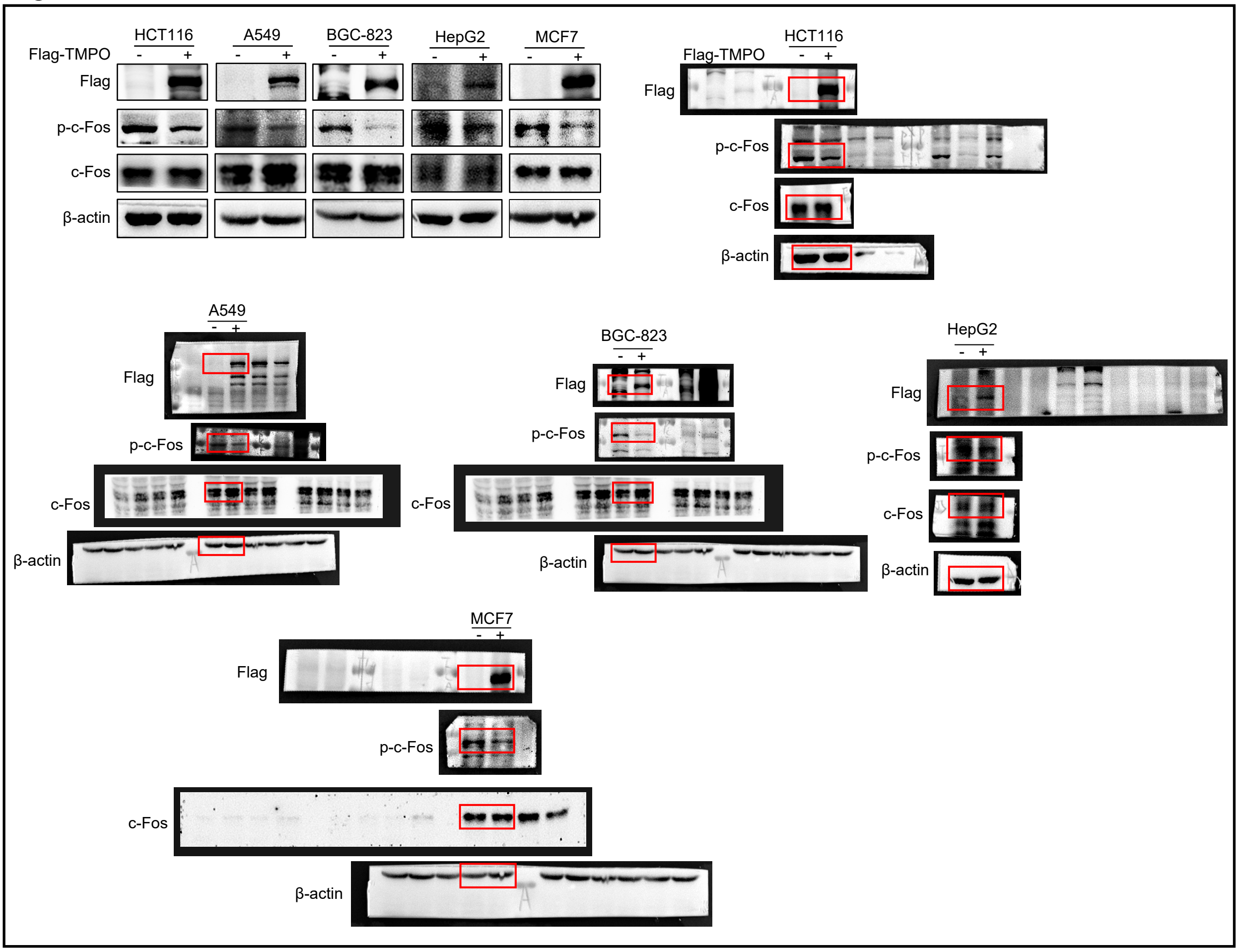

Figure 2D

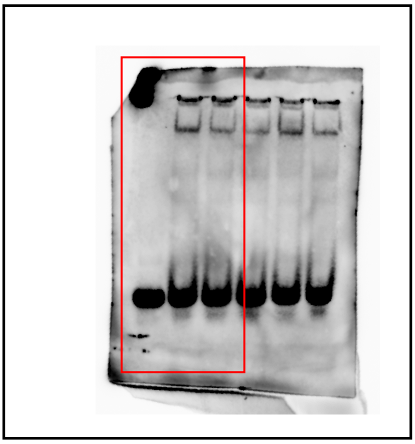

Figure 2E

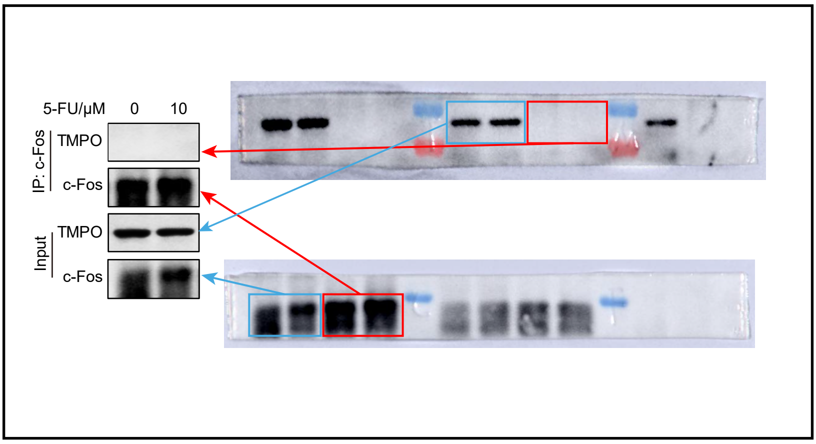

Figure 2F

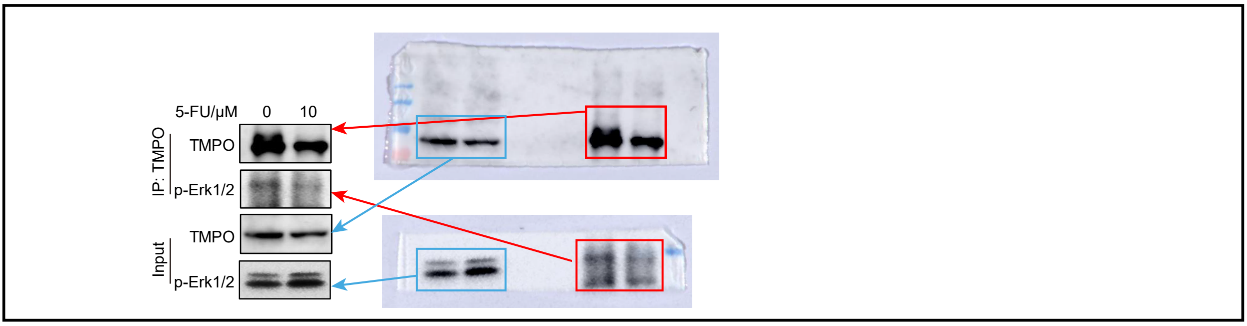

Full unedited gels for Figure 2H, 2I, and 2J

Figure 2H

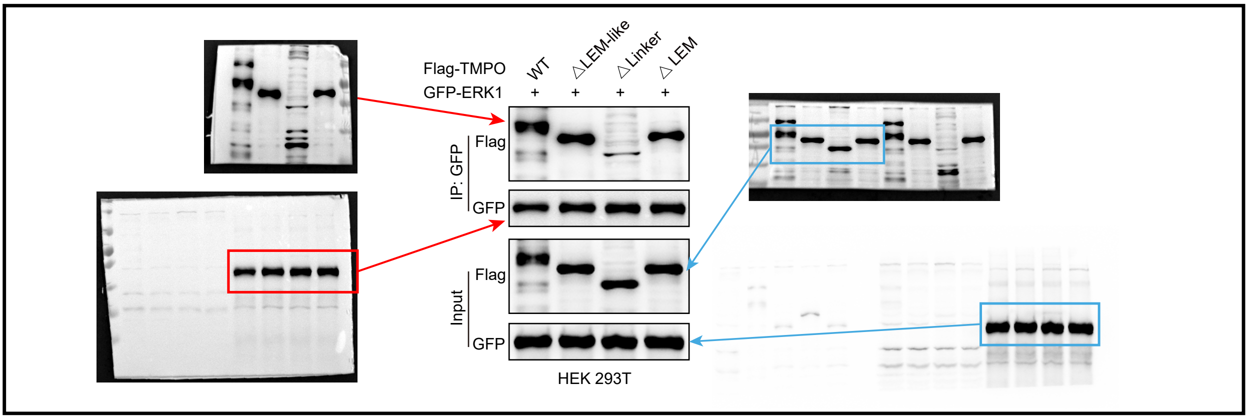

Figure 2I

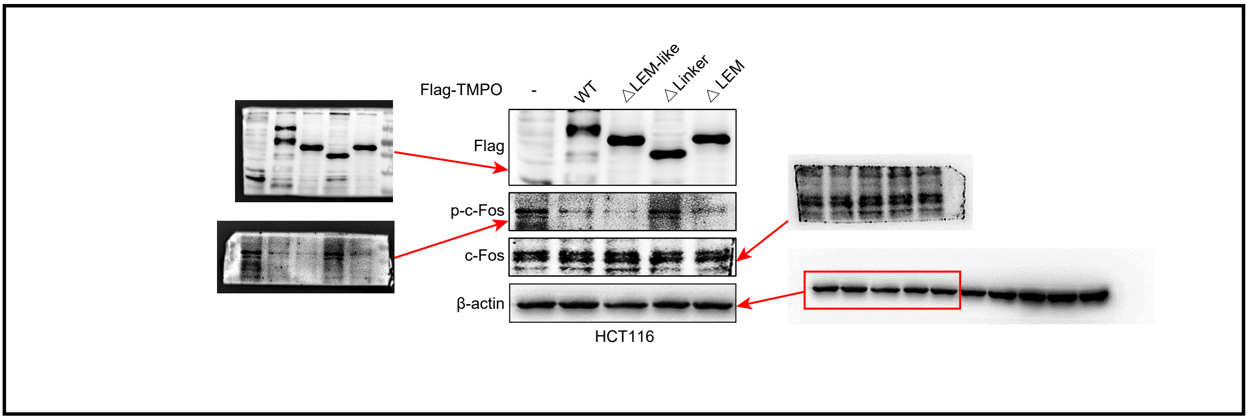

Figure 2J

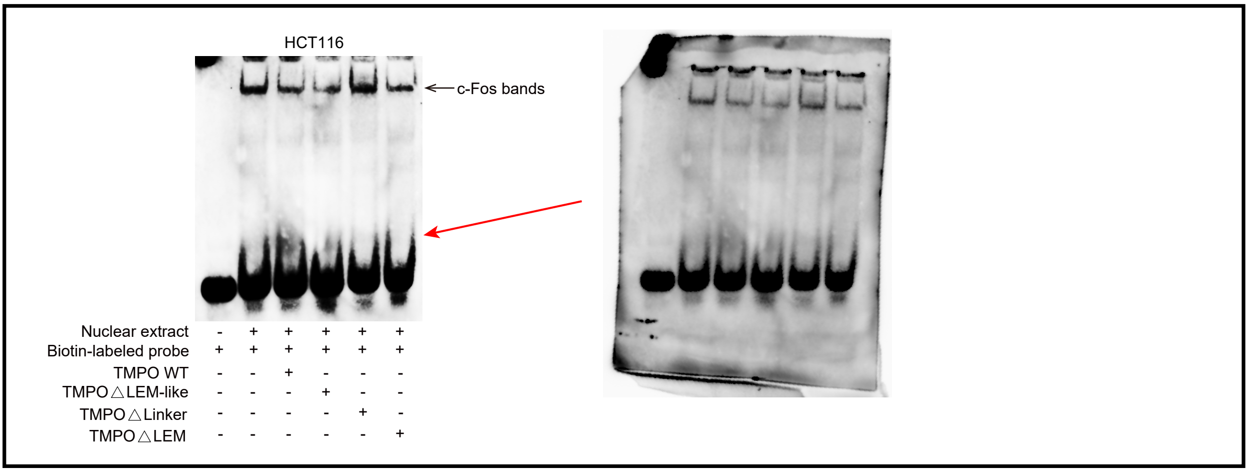

Full unedited gels for Figure 3A, 3B, 3C, 3D and 3E

Figure 3A

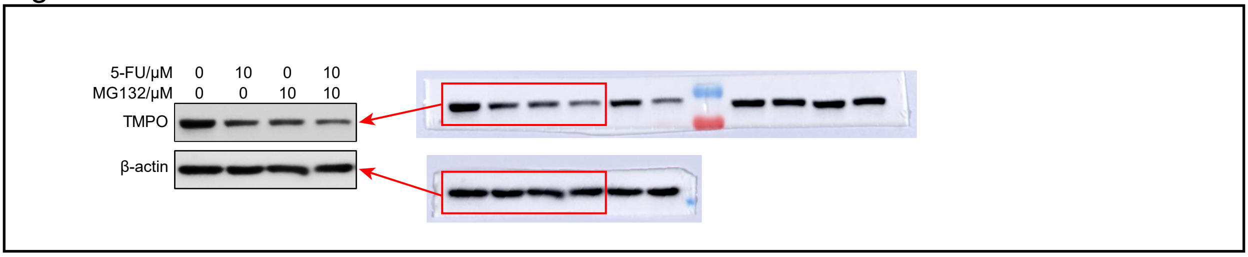

Figure 3B

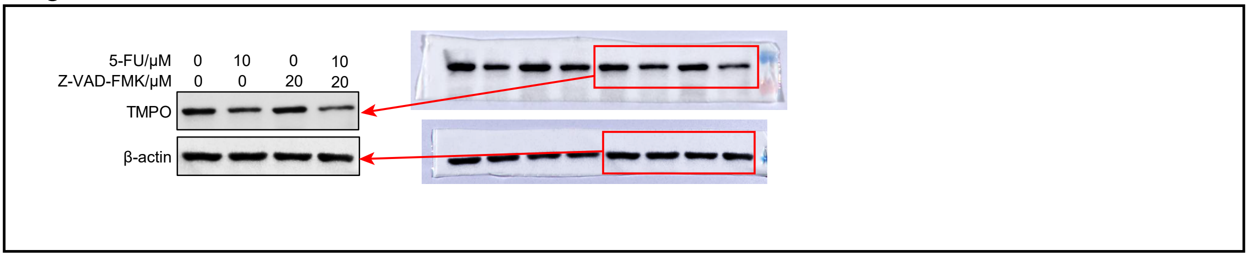

Figure 3C

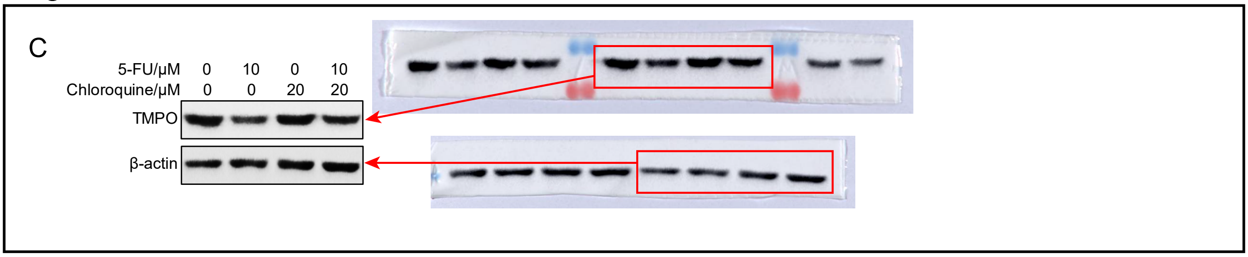

Figure 3D

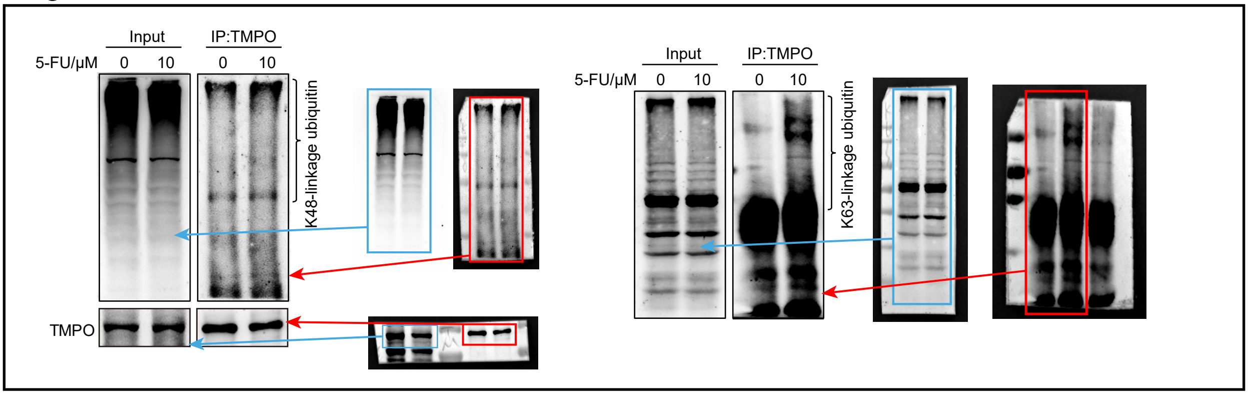

Figure 3E

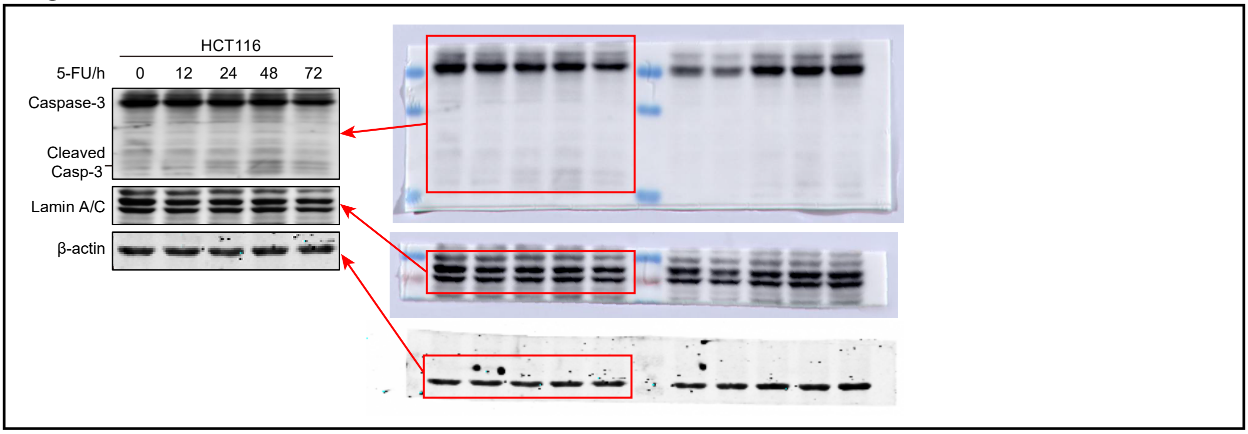

Full unedited gels for Figure 3F, 3G, 3H and 3I

Figure 3F

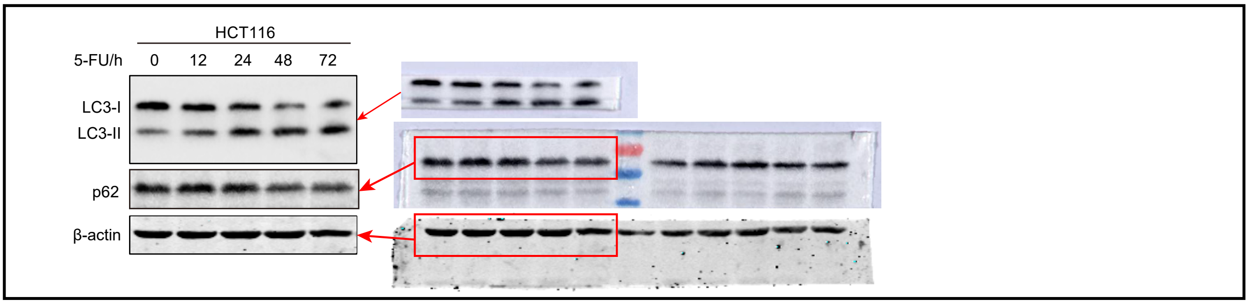

Figure 3G

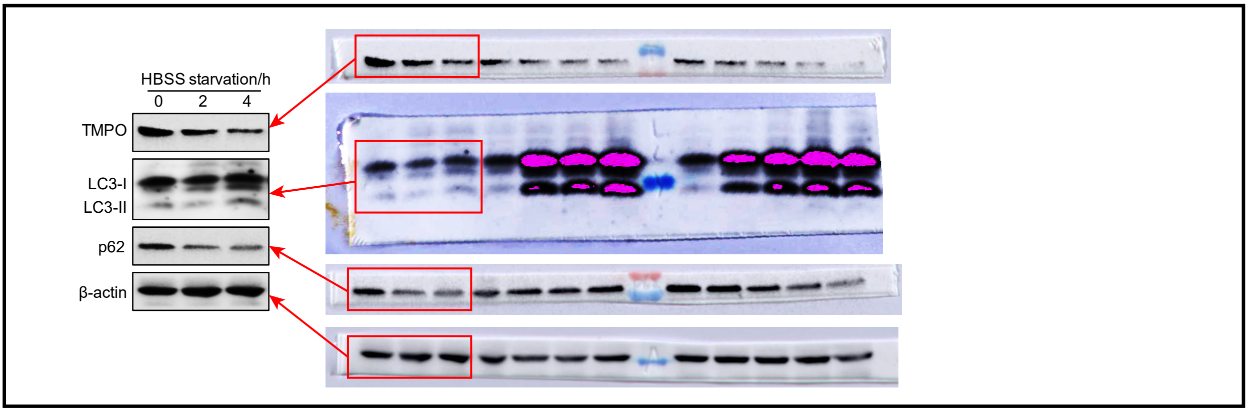

Figure 3H

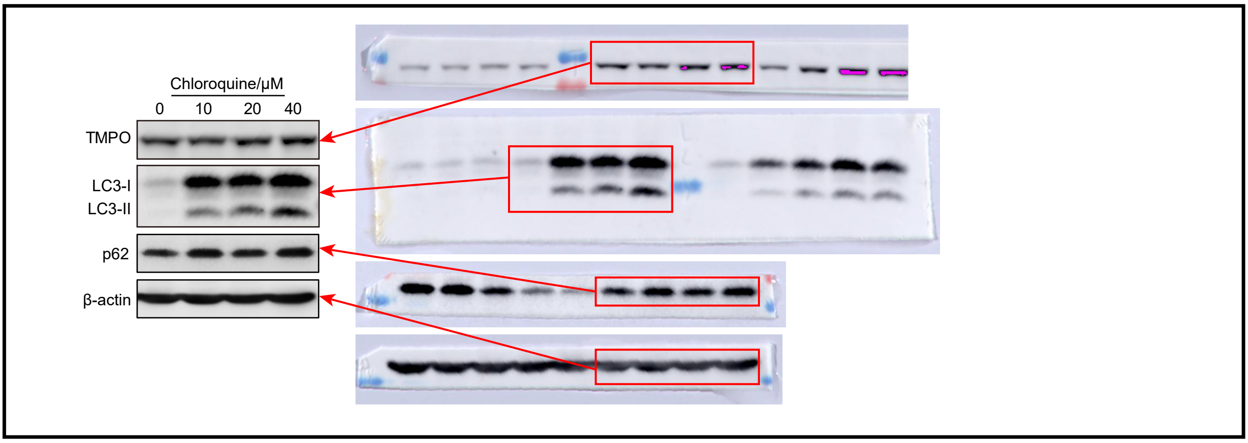

Figure 3I

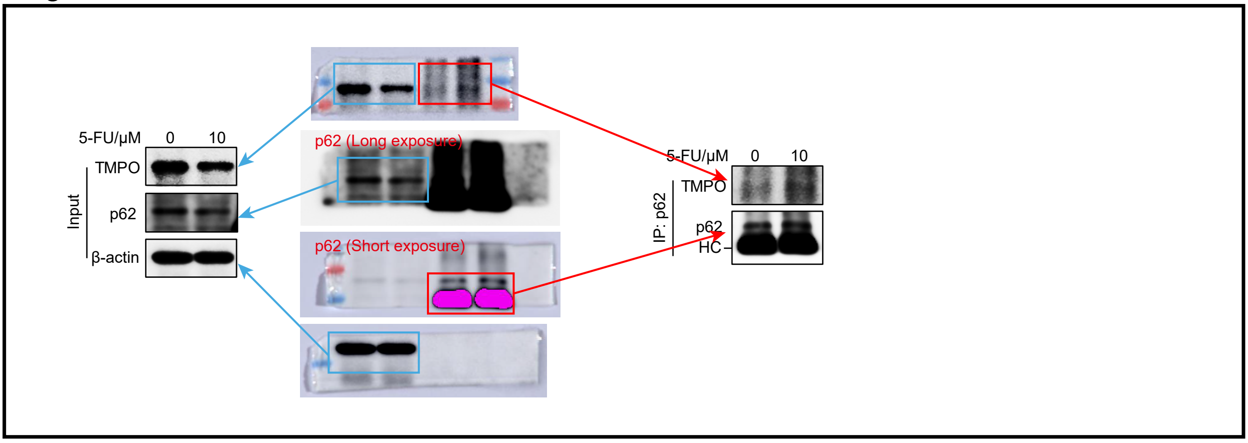

Full unedited gels for Figure 4E, 4F, 4G, 4L and 4M

Figure 4E

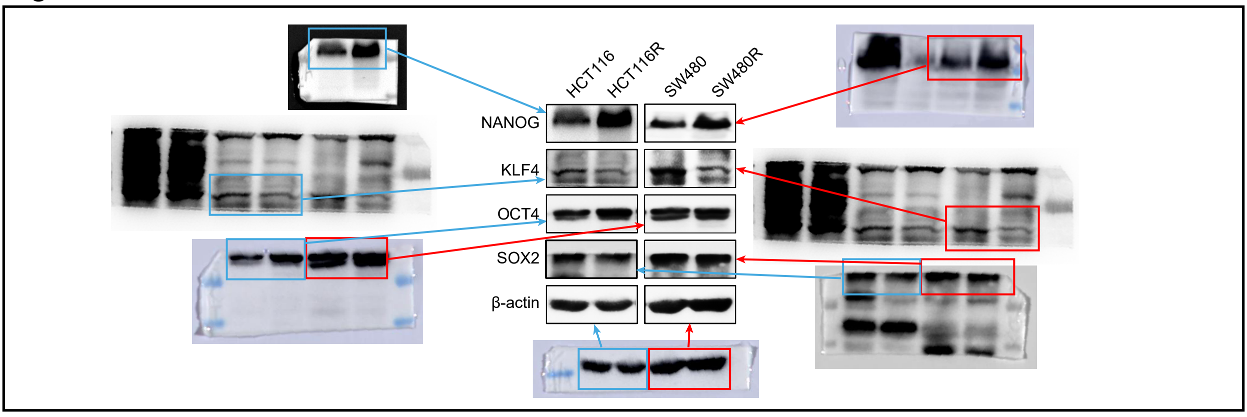

Figure 4F

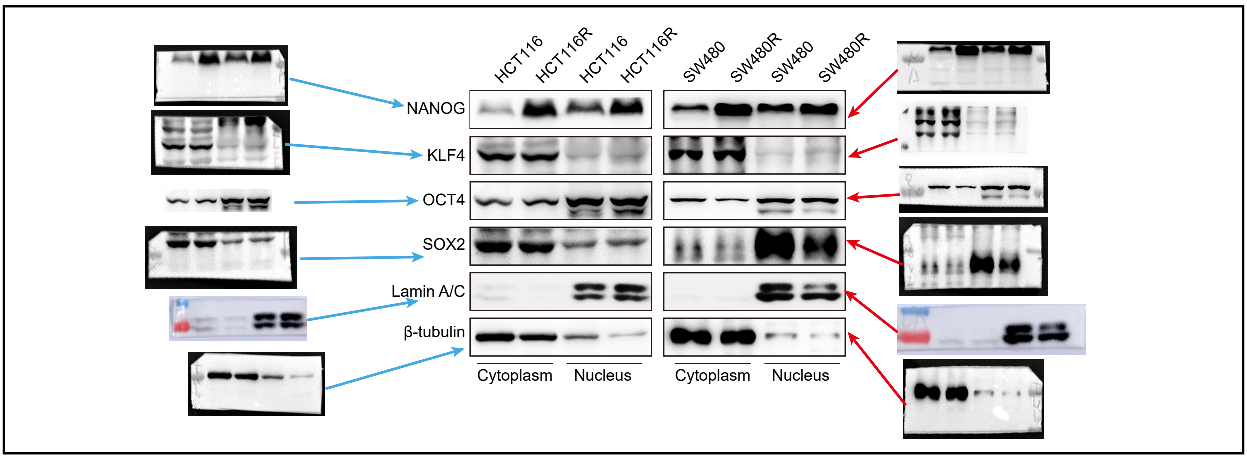

Figure 4G

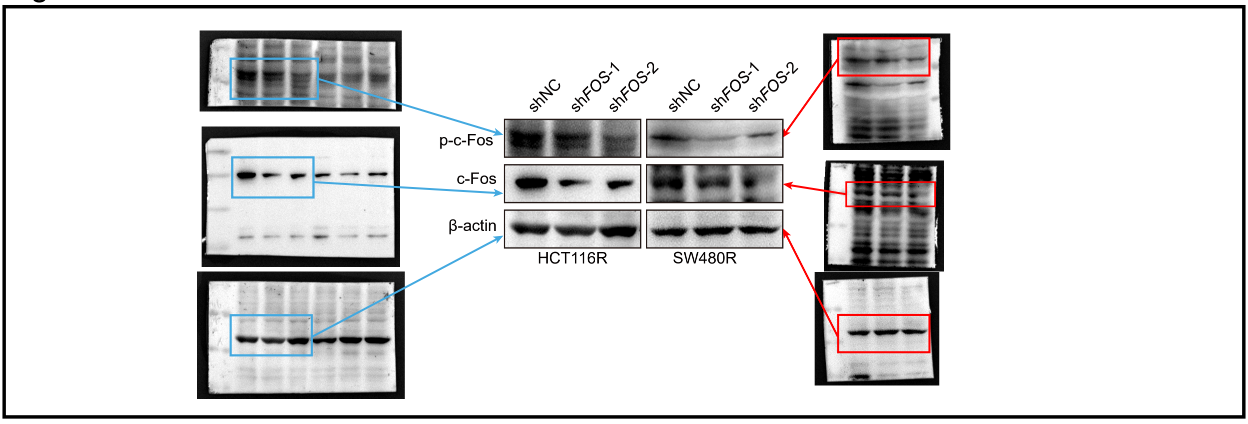

Figure 4M

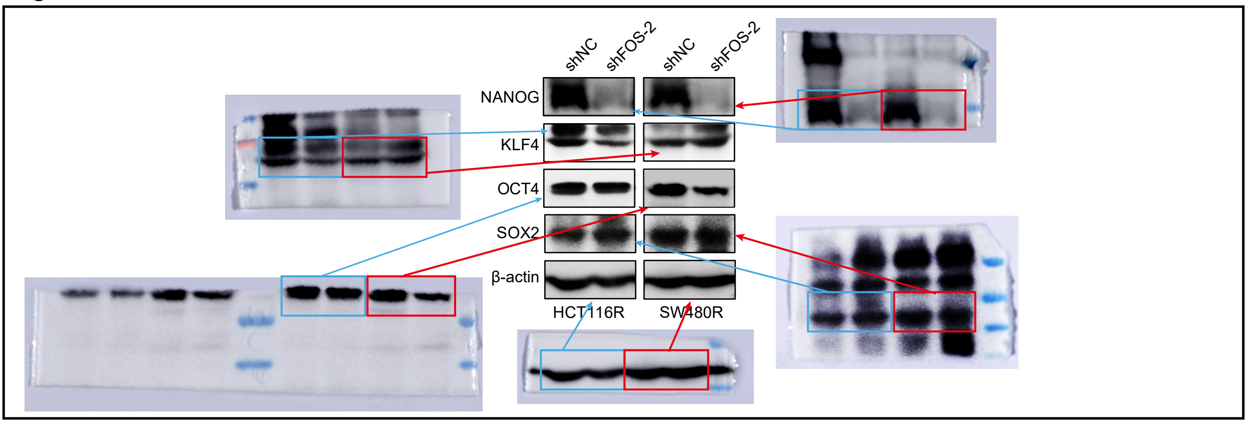

Figure 4N

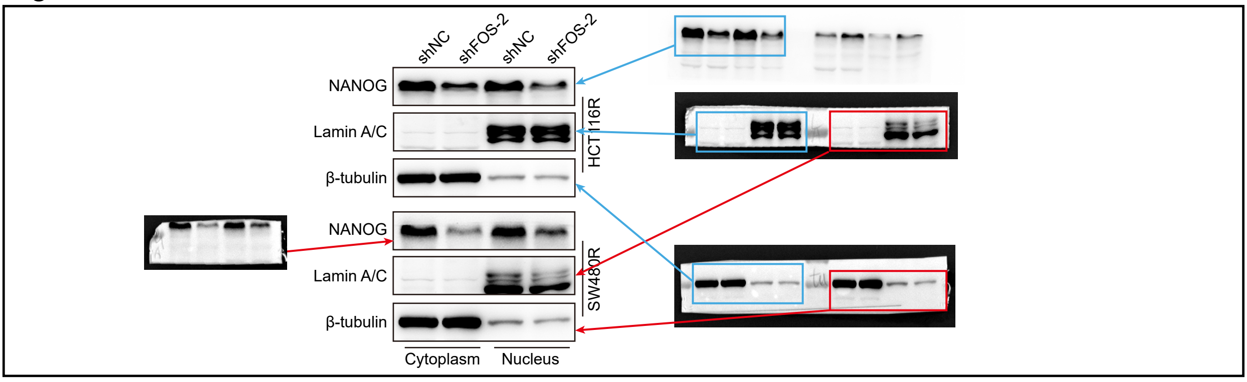

Full unedited gels for Figure 5A, 5I, 6G, and 7E

Figure 5A

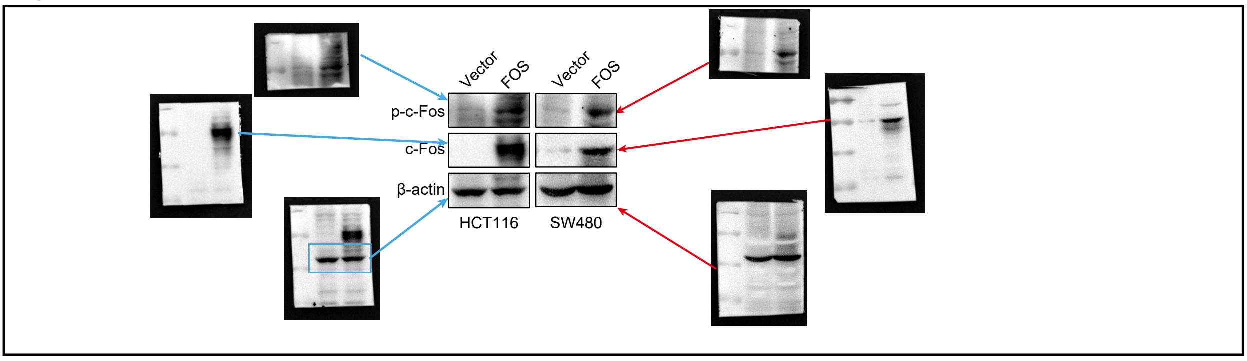

Figure 5I

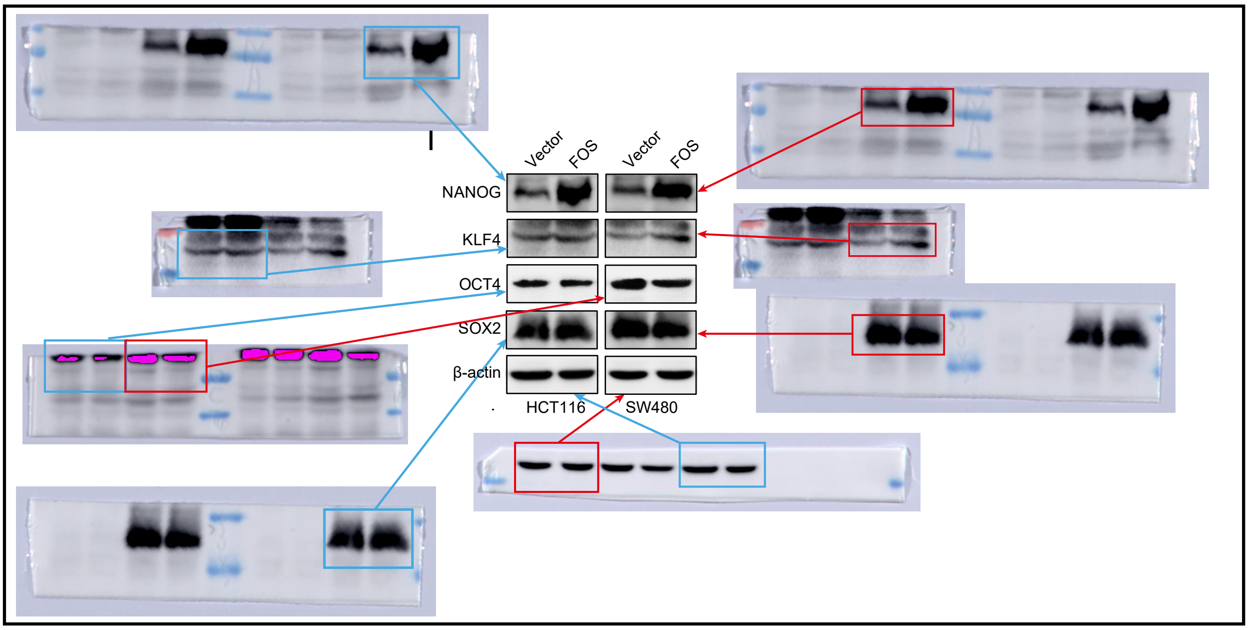

Figure 6G

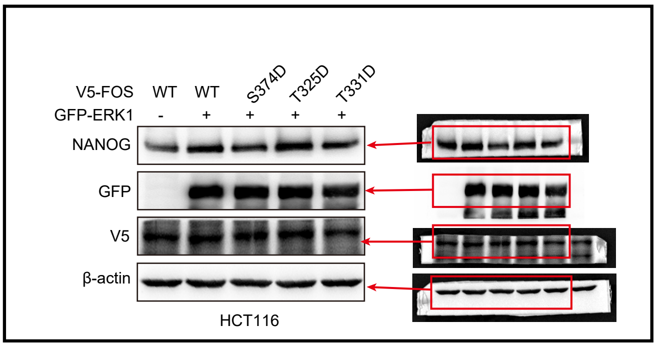

Figure 8E

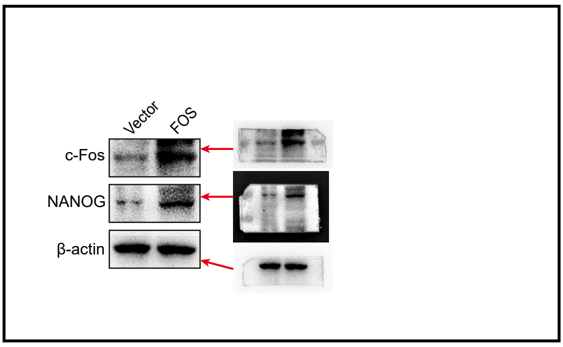

Figure 7A

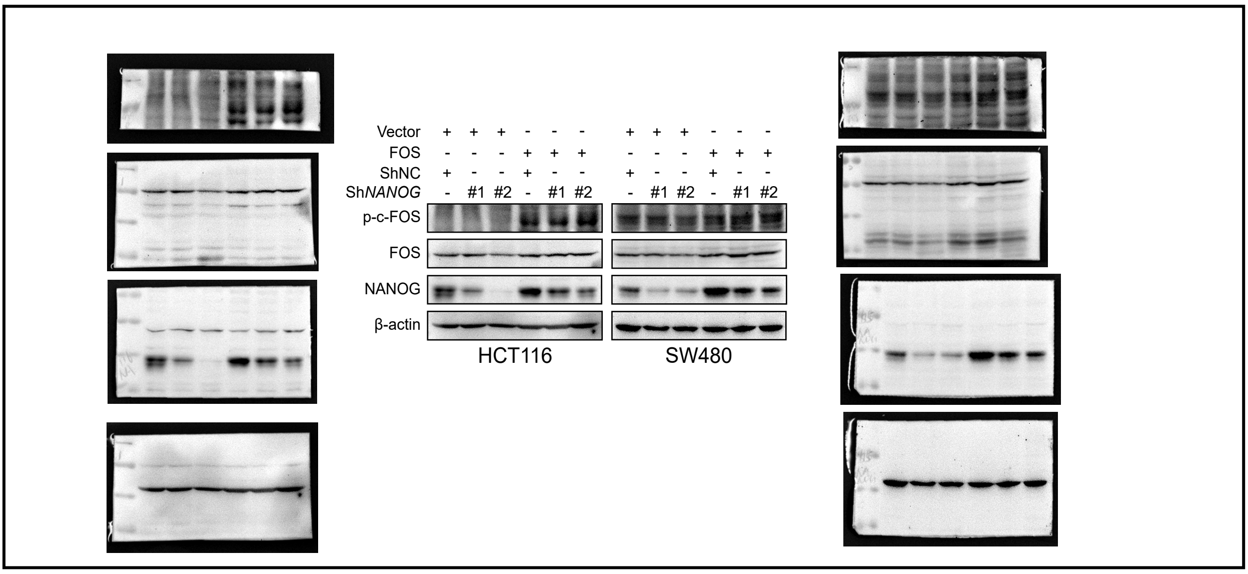

Full unedited gels for Supplementary Figure 2A

Figure S2A

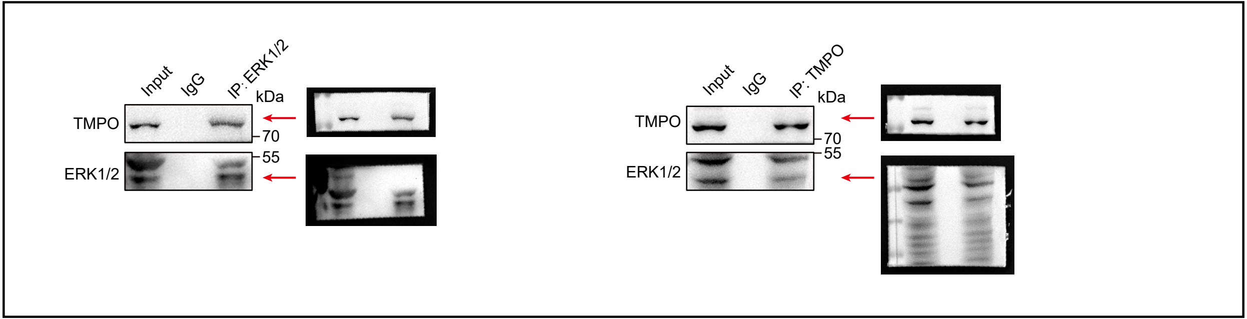

Supplement: Supplementary file 3 — Western blot original gels [file 41419_2024_6451_MOESM3_ESM.pdf]
